# Critical Review of Plant Cell Wall Matrix Polysaccharide Glycosyltransferase Activities Verified by Heterologous Protein Expression

**DOI:** 10.3389/fpls.2019.00915

**Published:** 2019-07-16

**Authors:** Robert A. Amos, Debra Mohnen

**Affiliations:** ^1^Complex Carbohydrate Research Center, University of Georgia, Athens, GA, United States; ^2^Department of Biochemistry and Molecular Biology, University of Georgia, Athens, GA, United States

**Keywords:** plant cell wall, glycosyltransferase, polysaccharide biosynthesis, heterologous expression, matrix glycan, enzyme mechanism, protein complex

## Abstract

The life cycle and development of plants requires the biosynthesis, deposition, and degradation of cell wall matrix polysaccharides. The structures of the diverse cell wall matrix polysaccharides influence commercially important properties of plant cells, including growth, biomass recalcitrance, organ abscission, and the shelf life of fruits. This review is a comprehensive summary of the matrix polysaccharide glycosyltransferase (GT) activities that have been verified using *in vitro* assays following heterologous GT protein expression. Plant cell wall (PCW) biosynthetic GTs are primarily integral transmembrane proteins localized to the endoplasmic reticulum and Golgi of the plant secretory system. The low abundance of these enzymes in plant tissues makes them particularly difficult to purify from native plant membranes in quantities sufficient for enzymatic characterization, which is essential to study the functions of the different GTs. Numerous activities in the synthesis of the major cell wall matrix glycans, including pectins, xylans, xyloglucan, mannans, mixed-linkage glucans (MLGs), and arabinogalactan components of AGP proteoglycans have been mapped to specific genes and multi-gene families. Cell wall GTs include those that synthesize the polymer backbones, those that elongate side branches with extended glycosyl chains, and those that add single monosaccharide linkages onto polysaccharide backbones and/or side branches. Three main strategies have been used to identify genes encoding GTs that synthesize cell wall linkages: analysis of membrane fractions enriched for cell wall biosynthetic activities, mutational genetics approaches investigating cell wall compositional phenotypes, and omics-directed identification of putative GTs from sequenced plant genomes. Here we compare the heterologous expression systems used to produce, purify, and study the enzyme activities of PCW GTs, with an emphasis on the eukaryotic systems *Nicotiana benthamiana, Pichia pastoris*, and human embryonic kidney (HEK293) cells. We discuss the enzymatic properties of GTs including kinetic rates, the chain lengths of polysaccharide products, acceptor oligosaccharide preferences, elongation mechanisms for the synthesis of long-chain polymers, and the formation of GT complexes. Future directions in the study of matrix polysaccharide biosynthesis are proposed.

## Introduction: Plant Cell Walls as Quality Factors in Fruit and Other Plant-Derived Commercial Products

Plants synthesize diverse cell wall polymers whose chemical structures contribute to the value of many plant-derived commercial products including wood, fibers and fabrics, thickening agents, drug delivery systems, and fruits. Plant cells are distinguished from other types of biomass by the presence of the plant cell wall (PCW), an extracellular matrix of polysaccharides and glycoproteins. The composition of PCWs varies among species and tissues. Cellulose and hemicellulosic polysaccharides can account for >60% of the dry mass of wood (Hoch, 2007). The fresh weight of tissues with high water content, such as apples, can consist of <10% cell wall material, the majority of which is pectic polysaccharides (Ng et al., 2013). The study of PCW polysaccharides is central to understanding the physical, chemical and textural properties that give value to plant products.

The deposition, crosslinking, remodeling, loosening, and degradation of matrix polysaccharides occurs in a dynamic manner that influences the progression of plant development (Drakakaki, 2015; Cosgrove, 2016a). The cell wall must have a structure strong enough to maintain cell integrity in response to internal turgor pressure but malleable enough to allow limited and directed cellular expansion (Cosgrove, 2016a). The cell wall affects most aspects of plant growth and morphology (Voxeur and Hofte, 2016), including changes that occur during fruit development and ripening. During the ripening process, for example, the cell wall loses structure due to degradation of matrix polysaccharides (pectin and hemicellulose) and loss of adhesion between polymers in the middle lamella (Ruiz-May and Rose, 2013; Paniagua et al., 2014). This controlled depolymerization of cell wall structure and loosening of cellular adhesion leads to the associated loss of fruit firmness and leakage of internal juices that occurs during the ripening process (Toivonen and Brummell, 2008; Paniagua et al., 2014; Dheilly et al., 2016).

Enzymes categorized as glycosyl hydrolases (GHs), lyases, and glycosidases catalyze the degradation of polysaccharide linkages. These families of degradative enzymes are secreted by plants and have been attractive candidates for mutational genetic studies to delay and control fruit softening. The down-regulation of two pectin-degrading genes, *polygalacturonase1* in strawberries and *pectate lyase* in tomatoes, both resulted in extended firmness, a phenotype that may be exploited to increase shelf life (Quesada et al., 2009; Kumar et al., 2014; Uluisik et al., 2016; Wang et al., 2018). The relationship between polygalacturonase1 secretion and apple softening was consistent with detection of the gene in “Royal Gala” apples, a cultivar that softens relatively rapidly, but not in “Scifresh” apples, whose flesh maintains increased tensile strength during ripening (Ng et al., 2013).

This review highlights progress made in the identification of the biosynthetic glycosyltransferases (GTs) that catalyze the formation of cell wall matrix polysaccharide linkages. The full network of genes associated with cell wall biosynthesis include cellulose and non-cellulosic GTs, lignin pathway biosynthetic enzymes, transcription factors, methyltransferases, acetyltransferases, wall structural proteins, extensins, expansins, and GHs involved in cell wall remodeling. Discovery of the functions of cell wall biosynthetic genes expands the possible targets for mutational genetic studies directed at breeding plants with favorable commercial properties. This review on genes encoding confirmed non-cellulosic glycosyltransferases exemplifies how the depolymerization of the crosslinked cell wall matrix polysaccharide network observed during organ abscission, senescence, and fruit ripening is made possible by the temporally directed process of cell wall biosynthesis.

## Models of Crosslinked Plant Cell Wall Polysaccharide Networks

During cellular division and formation of new plant cells, deposition of cell wall polysaccharides begins concurrent with the delivery of secretory lipids to form a cell plate (Drakakaki, 2015). Plant cells use different growth mechanisms, primarily diffuse growth observed in leaves (Cosgrove, 2018), linear diffuse growth observed in roots, and tip growth frequently represented by pollen tubes (Palin and Geitmann, 2012). These mechanisms share the coupling of cellular expansion with the secretion of matrix polysaccharides by the plant secretory system (Drakakaki, 2015; Van De Meene et al., 2017). Continued secretion of polysaccharides results in three PCW layers, in order of deposition: the middle lamella, the primary wall, and the secondary wall (Cosgrove, 2016b).

Pectic polysaccharides are deposited early during the formation of new cell walls during the process of cell plate formation (Daher and Braybrook, 2015; Drakakaki, 2015). As new layers of polysaccharides are secreted, internal pressure pushes the early layers outward, resulting in a pectin-rich middle lamella that forms an intercellular junction where water and solutes can freely diffuse. A standard view proposes that crosslinking of homogalacturonan (HG) within the middle lamella is a major factor in controlling cellular adhesion, and that loss of this crosslinking is a necessary step in organ abscission and fruit ripening (Daher and Braybrook, 2015).

The structural properties of the primary cell wall are influenced by all of the major polysaccharide components, as cellulose and hemicelluloses are also deposited during cell plate formation. The appearance of cellulose is associated with increased rigidity that occurs during maturation of the cell plate into distinct cell walls separating the adjacent daughter cells (Albersheim et al., 2010; Drakakaki, 2015). Order and toughness resulting from the deposition of cell wall polymers appears to increase during the later stages of cell wall formation, as the highly insoluble crystalline cellulose microfibrils are more prominently associated with the secondary wall deposited after the cell has stopped expanding (Albersheim et al., 2010; Cosgrove, 2014).

The layered, heterogeneous nature of PCWs has caused frequent debate and revisions to cell wall models in the literature. The cell wall controls cellular expansion by resisting internal turgor pressure, which is often referred to as the “load-bearing” function of cell walls that maintains cellular morphology (Cosgrove, 2016b). Based on the changing understanding of which matrix polysaccharides directly contact cellulose, various polysaccharide components have been assigned the role of “load-bearing” in different cell wall models. An influential early model proposed that the cellulose microfibrils are embedded within an interconnected non-cellulosic polysaccharide matrix, which effectively functions as a singular interconnected macromolecule (Keegstra et al., 1973; Cosgrove, 2016a). Due to growing evidence that pectins and hemicelluloses are not covalently linked to each other in a single matrix polysaccharide network, modified cell wall models began to emphasize that non-covalent interactions may be more important to the control of cell wall loosening and growth (Talbott and Ray, 1992). Later models proposed the existence of direct contacts between cellulose and xyloglucan (XG), formulating cellulose-XG as the load-bearing network, and pectin as a separate gel-like network (Carpita and Gibeaut, 1993; Cosgrove, 2016a). A complete model of how the cell wall creates a load-bearing network must account for all of the cross-linking interactions, a broad category of covalent and non-covalent contacts that provide strength to the cell wall.

Physical crosslinking of cellulose microfibrils is observable using high-resolution microscopy (Mccann et al., 1990). Cellulose-XG interactions may create adhesion zones that are resistant to depolymerization even though XG may only contact cellulose at limited surfaces (Dick-Perez et al., 2011; Park and Cosgrove, 2015; Cosgrove, 2016a). Xylan binds to the surface of microfibrils through hydrogen-bonding interactions (Busse-Wicher et al., 2014, 2016; Grantham et al., 2017), and antiparallel xylan chains may dimerize through GlcA-sidechain-mediated Ca^2+^ bridges (Pereira et al., 2017). The collection of crosslinking interactions also consists of direct covalent and glycosidic linkages between matrix polysaccharide domains and non-polysaccharide components of PCWs, including structural proteins and ferulic acid on lignin (Tan et al., 2013; De Oliveira et al., 2015).

Pectic polysaccharides also participate in PCW crosslinking interactions. Calcium ions mediate the formation of gel-like complexes between the carboxylic acid groups of GalA in HG chains (Grant et al., 1973; Morris et al., 1982; Cabrera et al., 2008). Because interaction with Ca^2+^ occurs through the anionic carboxylic acid group at O-6, these crosslinks can be blocked by methylation of this group, reduction of the Ca^2+^ concentration, or by the presence of chelators. Another pectic polysaccharide, RG-II, forms borate-mediated dimers through an apiose side chain diester, with possible contributions from other side chain monosaccharides such as l-Gal (Ridley et al., 2001; Sechet et al., 2018). Pectic polysaccharides appear to be spatially close to cellulose microfibrils (Wang et al., 2012; Wang and Hong, 2016; Broxterman and Schols, 2018) and possibly also XG (Park and Cosgrove, 2015). The chemical structures mediating pectin interactions with cellulose and XG have not been modeled, but the unexpected observation by solid-state NMR of potential cellulose-pectin contacts at a higher frequency than cellulose-XG contacts has been central to a growing interest in pectic polysaccharides as part of a single load-bearing network involving all cell wall polysaccharides, departing from earlier models (Wang et al., 2012; Cosgrove, 2016a; Wang and Hong, 2016).

Based on a model proposed for growth control in the algal species *Chara corallina*, HG-Ca^2+^ crosslinks potentially have a role in promoting cell expansion. As previously-deposited HG chains closely associated with cellulose microfibrils become load-bearing polymers in a stretched state, deposition of new HG may remove Ca^2+^ ions from older HG-Ca^2+^ structures, loosening inter-fibril interactions and priming the cell wall for turgor pressure-mediated expansion (Peaucelle et al., 2012). Uncertainty remains regarding the *in vivo* existence of calcium-linked pectin structures, particularly in the outer layers of cell walls within the apoplast (Hocq et al., 2017; Voiniciuc et al., 2018b). Deposition of de-esterified pectin occurs in root hair tips, which is inconsistent with the model presented by pollen tube tips, in which HG is deposited in a non-crosslinked methyl-esterified form (Mravec et al., 2017). However, chelation of calcium causes cellular separation (Daher and Braybrook, 2015), and downregulation of the pectin biosynthetic gene GAUT4 in transgenic switchgrass causes a reduction in total cell wall calcium (Biswal et al., 2018). These observations support HG-Ca^2+^ as important for growth and cellular adhesion, as proposed in standard models.

The chemical composition of cell wall polysaccharides is a mature area of study, and composite models of how these polysaccharides interact and function continue to grow more sophisticated. An ongoing challenge remains to discover the GTs that synthesize the cell wall and to study their enzymatic properties using biochemical assays.

## Strategies for Identifying Cell Wall Glycosyltransferase (CWGT) Activities

Plant cell wall matrix polysaccharides are synthesized intracellularly and exported by post-Golgi vesicular transport in the secretory pathway (Kim and Brandizzi, 2014). GTs localized to the Golgi apparatus produce a distinct punctate pattern and co-localize with Golgi markers when transiently expressed as fusion proteins with green fluorescent protein (Boevink et al., 1998; Atmodjo et al., 2011). The correlation of this fluorescent punctate pattern with Golgi localization has been confirmed with higher resolution images obtained using immunogold labeling, which visualizes individual Golgi stacks (Zhang and Staehelin, 1992; Boevink et al., 1998).

Prior to the identification of individual GTs, biosynthetic activities were measured using microsomal membrane fractions enriched for Golgi-localized enzymes. ER-and-Golgi-enriched microsomal membrane fractions retain integral membrane proteins following centrifugation (Fujiki et al., 1982). These techniques have been used to measure the biosynthesis of PCW matrix polysaccharides since at least the 1960s, with the measurement of Golgi membrane-linked synthesis of the α-1,4-galacturonic acid backbone of HG (Villemez et al., 1965) and the β-1,4-glucan backbone of XG (Ray et al., 1969).

**Figure 1:**
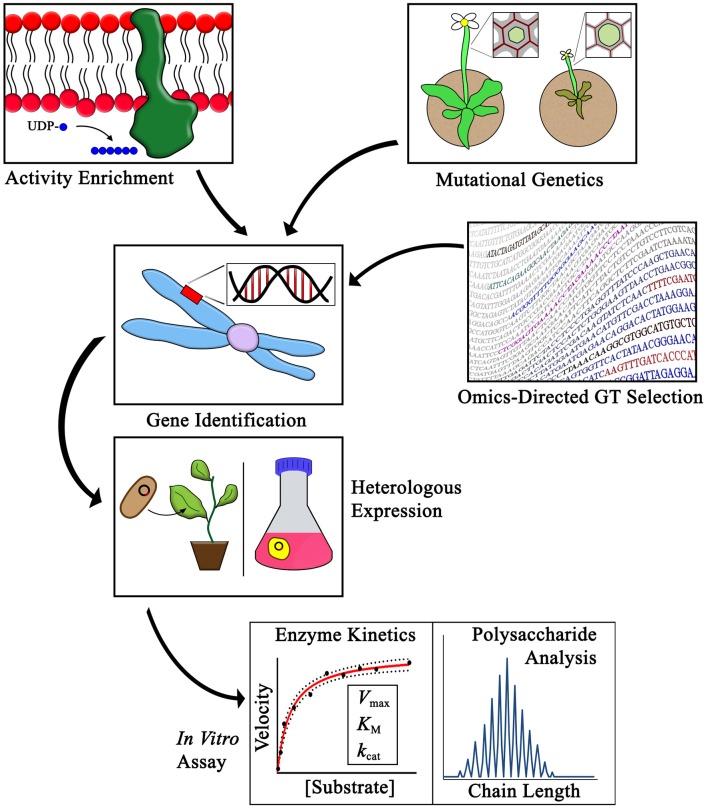
Methods for identifying CWGTs for *in vitro* activity verification. The activities of plant cell wall glycosyltransferases have been identified using three main strategies as outlined in this review: activity enrichment, mutational genetics, and omics-directed GT selection. Heterologous expression and *in vitro* assays are critical steps in the process of verifying the synthesis of specific cell wall linkages associated with different families of CWGTs. Purification of enzymes following heterologous expressional enables advanced studies of GT mechanism, including enzyme kinetic, and polysaccharide chain length analyses.

Various strategies have been developed with the goal of identifying putative CWGTs so that they can be expressed and studied as purified recombinant proteins. These strategies fall into three broad categories: activity enrichment, mutational genetics, and omics-directed GT selection. A complementary gain-of-function assay method has also been used as an *in vivo* system to detect synthesis of polysaccharides not normally found in Arabidopsis. The general outline of using these strategies to identify CWGTs is depicted in Figure 1. The first uses of heterologously expressed enzymes to demonstrate the synthesis of specific PCW linkages by proteins encoded by proposed CWGT genes were completed in 1999 with the identification of the XG side-chain fucosyltransferase FUT1 (Perrin et al., 1999) and a galactomannan-specific galactosyltransferase (Edwards et al., 1999).

Several factors contributed to the acceleration of GT identification after 1999. The Arabidopsis genome sequence was published and made available in 2000 (Arabidopsis Genome Initiative, 2000) and the Arabidopsis Information Resource has been continually maintained as a database of all annotated Arabidopsis genes since then (Berardini et al., 2015). Lacking complete genomes, some of the early studies mapped gene sequences to expressed sequence tag libraries (Perrin et al., 1999; Dhugga et al., 2004) or to protein sequence data (Edwards et al., 1999). The Carbohydrate Active Enzymes database (CAZy) was also developed which serves as an essential resource for studies of carbohydrate-acting enzymes. The CAZy database groups characterized and putative GTs and other carbohydrate-active enzymes into families with shared catalytic, mechanistic, and structural characteristics (Lombard et al., 2014). Availability of these gene databases enhanced the power of high-throughput screening methods, simplifying the process of identifying putative GTs using proteomic and bioinformatic methods.

The families of PCW matrix polysaccharide glycosyltransferases (CWGTs) of known activity, identified following heterologous expression, are summarized in Tables 1–**6**. Many more genes exist for which mutational genetics and other methods have provided data in support of proposed putative activities. For the purposes of this review, the focus has been limited to PCW GTs for which enzyme activity has been confirmed using *in vitro* assays.

**Table 1:** Xyloglucan biosynthetic GTs of known function determined by heterologous expression.

**Xyloglucan (XG)**							
**Enzyme or family**	**Sugar trans.^a^**	**Activity^b^, ***Product synthesized***^c^**	**GT^d^**	**Acceptor^e^**	**Activity notes**	**Homology/redundancy (Arabidopsis)**	**References**
CSLC	D-Glc	β-1,4-Glc *XG Backbone*	2	Unknown: endogenous acceptors or *de novo* synthesis in *P. Pastoris*.	Unknown elongation size: limited solubility of β-glucan oligosaccharides with chain lengths larger than DP 6. High MW polymerization unknown.	Five-member family. Activity for CSLC4 only.	Cocuron et al., 2007; Davis et al., 2010
XXT	D-Xyl	α-1,6-Xyl side chain initiation on XG backbone *X Side chain*	34	XG backbone oligosaccharides, DP 4–6. DP 3 acceptor tested, no activity detected.	Single addition to GGGGGG synthesizes GGXGGG. Less efficient second product, GGXXGG. DP ≥ 4 acceptor required for activity. DP 6 acceptor preferred to DP 5.	Five-member family. Activity for XXT1, 2, 4, and 5.	Faik et al., 2002; Cavalier and Keegstra, 2006; Vuttipongchaikij et al., 2012; Culbertson et al., 2016, 2018; Ruprecht et al., 2018
MUR3	D-Gal	β-1,2-Gal addition to X side chain (Xyl residue transferred by XXT) *L Side chain*	47	XG oligosaccharides extracted from *mur3*-deficient plants with acceptor sites for Gal transfer, unknown size/DP.	Single addition to XXXG synthesizes XXLG.	One homolog: XLT2. Activity predicted from mutant phenotype.	Madson et al., 2003
FUT1 *MUR2*	L-Fuc	α-1,2-Fuc addition to L side chain (Gal residue transferred by MUR3) *F side chain*	37	XG oligosaccharides with acceptor sites for Fuc transfer, DP 4: XXLG or XLLG.	Single addition to XXLG synthesizes XXFG. Single addition to XLLG synthesizes XLFG.	10-member FUT family. XG-related activity for FUT1 only. FUT4 and FUT6 transfer Fuc to AGP side chains.	Perrin et al., 1999; Faik et al., 2000; Vanzin et al., 2002; Ciceron et al., 2016; Rocha et al., 2016; Urbanowicz et al., 2017

*Abbreviated gene annotations are used (e.g., CSLC rather than “Cellulose synthase-like C”). Additional annotations found in the literature are listed in italics below the gene name. Standard abbreviations for monosaccharides are used. All acceptors are standardized to degree of polymerization (DP) for chain length formatting (e.g., xylohexaose (Xyl_6_) is listed as “Xylan backbone oligosaccharides, DP 6”). Listed references are original research publications in which the enzyme (or enzyme family) was heterologously expressed or review articles that contain additional information listed in the table*.

a *Trans. is an abbreviation for transferred*.

b *Activities are listed as simplified descriptions of the monosaccharide transferred. Side chain activities are listed as “initiation” for the first residue attached to the polysaccharide backbone or “addition” for subsequent residues added to an extended side chain. Full activity names include the designation “transferase” and the polysaccharide acceptor, where applicable. As an example, the full activity name of XG backbone synthesis is xyloglucan:β-1,4-glucosyltransferase (XG:β-1,4-GlcT) and the full linkage synthesized is Glcβ-1,4-Glc*.

c *Products synthesized are listed in italics by common names used in the literature, where applicable*.

d *CAZy GT family*.

e *Only acceptors with confirmed activity in the references cited are listed. Other acceptors that did not yield activity may have been tested and are not included*.

### Activity Enrichment

A long-discussed problem in glycobiology is the difficulty of identifying Golgi-localized GTs due to their low abundance (Fukuda et al., 1996; Sandhu et al., 2009). The first plant CWGT mapped to an activity, FUT1, was estimated to be <0.01% of total cellular protein (Faik et al., 2000; Table 1). Activity enrichment requires an activity assay that can detect transfer of a sugar in a specific anomeric configuration and glycosyl linkage using proteins present in microsomal membrane preparations. Incubation of a microsomal enzyme source with nucleotide sugar donors, which are often radiolabeled to enhance detection sensitivity, can lead to incorporation of products into either exogenously added oligosaccharide acceptors or onto endogenous acceptors within the microsomes.

Because the composition of cell wall polysaccharides is different across species, tissues, and developmental stages, techniques have taken advantage of tissues enriched for a specific CWGT activity. The mannan synthase that synthesizes the β-1,4-mannose backbone of galactomannan (GM) (CSLA9 in Arabidopsis) and the galactosyltransferase (GalT) that adds α-1,6-galactose side chains to the mannan backbone were identified using guar and fenugreek seed endosperms, respectively (Table 3). Because some legume seed endosperms produce large amounts of GM as a storage polysaccharide in early developmental stages, GM biosynthetic activities and transcription for these two genes was found to be highly upregulated in these tissues. N-terminal sequencing of peptides within the activity-enriched fraction identified the putative GTs (Edwards et al., 1999; Dhugga et al., 2004). A similar technique was used to identify the glucan synthase that synthesizes the β-1,4-linked backbone of XG (CSLC4 in Arabidopsis), because nasturtium seeds deposit large amounts of XG as a storage polysaccharide (Cocuron et al., 2007; Table 1).

Synthesis of the homogalacturonan backbone, α-1,4-linked GalA, has been detected using microsomal preparations from varied plant sources, including pea (Villemez et al., 1965), tobacco (Doong et al., 1995), Azuki bean (Yasui et al., 2009), and *petunia axillaris* (Akita et al., 2002). Sequential purification led to a semi-purified Arabidopsis membrane fraction enriched for proteins with the predicted GT sequences for GAUT1 and GAUT7. Activity was traced over multiple purification steps using an assay in which GalA was transferred from UDP-GalA onto oligogalacturonide acceptors (Sterling et al., 2006; **Table 5**). Recently, development of an assay to measure the transfer of rhamnose from UDP-Rha onto acceptors with the [-4-GalA-α-1-2-Rha-α-1-] disaccharide backbone-repeat of rhamnogalacturonan-I (RG-I) enabled the detection of RG-I biosynthetic activity in Azuki bean epicotyls (Uehara et al., 2017; **Table 5**). Because RG-I is upregulated in Arabidopsis seed coat mucilage, the putative RG-I rhamnosyltransferase RRT1 was identified as a GT that was downregulated following knockdown of two mucilage-related transcription factors (Takenaka et al., 2018).

### Mutational Genetics

A second strategy is mutational genetics, in which CWGTs are identified due to cell wall-specific phenotypes resulting from mutations in unique GT sequences within the genome. Because the monosaccharide linkages of cell wall polysaccharides are known, deficiency in a particular monosaccharide linkage in a mutant is generally hypothesized to be due to a mutation in an enzyme associated with the synthesis of that linkage.

In a landmark study, 11 genes were identified in a screen of 5,200 mutagenized plants by selecting for strong losses or changes in cell wall monosaccharide composition. The mutants were annotated MUR1-MUR11 (Reiter et al., 1997). Two of the genes identified, MUR2 (FUT1) and MUR3 have been mapped to XG backbone biosynthetic functions. The *mur2* mutation was strongly deficient in cell wall fucose, specifically XG fucosylation (Reiter et al., 1997; Vanzin et al., 2002). FUT1 was independently identified by an activity enrichment method using solubilized pea epicotyls (Perrin et al., 1999; Table 1), and these activity results were consistent with the fucose defect caused by the mutant (Vanzin et al., 2002). The mutant *mur3*, like the mutant *mur2*, was also originally identified as deficient in fucose based on analyses of the total cell walls of Arabidopsis leaves (Reiter et al., 1997). Upon fractionation of *mur3* leaves, hemicellulose-enriched fractions were found to be deficient in both fucose and galactose. MUR3 was shown to encode the galactosyltransferase that adds galactose to make the L side chain of XG. Although MUR3 is a GalT, the fucose deficiency resulted from the lack of the acceptor Gal onto which FUT1 transfers fucose to create the F side chain (Madson et al., 2003; Table 1).

Another series of mutants, the *irregular xylem* or *irx* mutants, have proposed functions in cell wall synthesis. The irregular xylem phenotype was originally described in three mutants, annotated *irx1-irx3*, based on a collapsed xylem phenotype visible in cross-sections of stem vascular tissues. This phenotype was predicted to be relevant to the loss of secondary cell wall polysaccharides necessary to maintain cell wall integrity during water transport and transpiration (Turner and Somerville, 1997). Additional mutations yielding the irregular xylem phenotype have been visualized (Brown et al., 2005), leading to the identification of IRX10-L and IRX10 GTs that synthesize the β-1,4-linked xylose backbone of xylan (Jensen et al., 2014; Urbanowicz et al., 2014; Table 2). Four related proteins with a similar mutant phenotype, IRX9, IRX9-L, IRX14, and IRX14-L, are putative xylan biosynthetic GTs, but their activities have not been confirmed *in vitro* (Smith et al., 2017).

**Table 2:** Xylan biosynthetic GTs of known function determined by heterologous expression.

**Xylan**							
**Enzyme or family**	**Sugar trans.^a^**	**Activity^b^**, ***Product synthesized*****^c^**	**GT^d^**	**Acceptor^e^**	**Activity notes**	**Homology/redundancy (Arabidopsis)**	**References**
XYS1 *IRX10-L*	D-Xyl	β-1,4-Xyl *Xylan Backbone*	47	Xylan backbone oligosaccharides, DP 2–6. Xyl monosaccharide tested, no activity detected.	Elongation of DP 6 acceptor up to DP ~21 detected by MALDI. High MW polymerization unknown.	One homolog, IRX10 with similar activity. Two related GT43 enzymes (IRX14 and IRX14-L) are putative xylan backbone transferases. Activity not confirmed.	Zeng et al., 2010, 2016; Lee et al., 2012b; Jensen et al., 2014, 2018; Urbanowicz et al., 2014; Jiang et al., 2016
GUX	D-GlcA	α-1,2-GlcA side chain initiation on xylan backbone	8	Xylan backbone acceptors, DP 2–6. Xyl monosaccharide tested, no activity detected.	Single addition to DP 6 acceptor. Fifth Xylose from nonreducing end preferred (XXXXGX). Some product contains single addition on third Xylose from nonreducing end (XXGXGX). Strong preference for longer acceptors (DP ≥ 6).	Five-member family. Activity for GUX1, 2 and 4. GUX3 activity inconsistent from two independent publications: GUX3 and GUX5 may be noncatalytic.	Lee et al., 2012a; Rennie et al., 2012
XAT1	L-Ara	α-1,3-Ara side chain initiation on xylan backbone	61	Unknown: endogenous xylan acceptors in Arabidopsis. Gain-of-function evidence only.	Single addition to xylan acceptors of unknown size. Digested products reveal a pattern of DP 5 products containing an Ara side chain on the second Xyl from the nonreducing end (XAXX).	Wheat/rice gene. Arabinoxylans are low-abundance in Arabidopsis. Two putative GT61 homologs.	Anders et al., 2012
XAX1	D-Xyl	β-1,2-Xyl addition to α-1,3-linked Ara side chain (residues transferred by XAT1) on grass xylan	61	Unknown: endogenous xylan acceptors in *N. benthamiana*.	Unknown product size/structure: Most likely product detected by NMR is a xylan backbone oligosaccharide, DP 4, containing a Xyl-Ara side chain on the second Xyl from the nonreducing end.	Rice gene. Grass-specific clade not expected to be found in Arabidopsis.	Chiniquy et al., 2012

*Gene annotations, abbreviations, and references are formatted as in Table 1*.

a *Trans. is an abbreviation for transferred*.

b *Activities are listed as simplified descriptions of the monosaccharide transferred. Side chain activities are listed as “initiation” for the first residue attached to the polysaccharide backbone or “addition” for subsequent residues added to an extended side chain. Full activity names include the designation “transferase” and the polysaccharide acceptor, where applicable. As an example, the full activity name of xylan backbone synthesis is xylan:β-1,4-glucosyltransferase (xylan:β-1,4-GlcT) and the full linkage synthesized is Glcβ-1,4-Glc*.

c *Products synthesized are listed in italics by common names used in the literature, where applicable*.

d *CAZy GT family*.

e *Only acceptors with confirmed activity in the references cited are listed. Other acceptors that did not yield activity may have been tested and are not included*.

**Table 3:** Heteromannan biosynthetic GTs of known function determined by heterologous expression.

**Mannan/Glucomannan (GM)**							
**Enzyme or family**	**Sugar trans.^a^**	**Activity^b^, ***Product synthesized***^c^**	**GT^d^**	**Acceptor^e^**	**Activity notes**	**Homology/redundancy (Arabidopsis)**	**References**
CSLA	D-Man	β-1,4-Man and β-1,4-Glc linkages *Glucomannan (GM) backbone*	2	Unknown: endogenous acceptors or *de novo* synthesis in *Drosophila* S2 cells and in *P. Pastoris*.	Synthesizes β-1,4-linked polymers containing Glc, Man, or GlcMan depending on substrate availability. High MW products (>100 kDa) detected by size exclusion.	Nine-member family. Activity for CSLA2, 7, and 9. Mutational evidence for CSLA3 activity.	Dhugga et al., 2004; Liepman et al., 2005; Goubet et al., 2009; Voiniciuc et al., 2019
CSLD	D-Man	β-1,4-Man *Mannan backbone*	2	Unknown: endogenous acceptors or *de novo* synthesis in *N. benthamiana*.	Unknown elongation size. High MW polymerization unknown.	Six-member family. Activity for CSLD5 and co-expressed CSLD2:CSLD3, but not CSLD2 or CSLD3 individually.	Verhertbruggen et al., 2011; Yin et al., 2011
GalT	D-Gal	α-1,6-Gal side chain initiation on mannan backbone	34	β-1,4-Man mannan backbone oligosaccharides DP 5–10. DP 1–4 acceptors tested, no activity detected.	Single addition to mannan backbone. Unknown regiospecificity of addition. Addition to glucomannan backbone not confirmed *in vitro*.	Fenugreek enzyme. Putative Arabidopsis homolog MUCI10.	Edwards et al., 1999; Voiniciuc et al., 2015

*Gene annotations, abbreviations, and references are formatted as in Table 1*.

a *Trans. is an abbreviation for transferred*.

b *Activities are listed as simplified descriptions of the monosaccharide transferred. Side chain activities are listed as “initiation” for the first residue attached to the polysaccharide backbone or “addition” for subsequent residues added to an extended side chain. Full activity names include the designation “transferase” and the polysaccharide acceptor, where applicable. As an example, the full activity name of mannan backbone synthesis is mannan:β-1,4-mannosyltransferase (mannan:β-1,4-ManT) and the full linkage synthesized is Manβ-1,4-Man*.

c *Products synthesized are listed in italics by common names used in the literature, where applicable*.

d *CAZy GT family*.

e *Only acceptors with confirmed activity in the references cited are listed. Other acceptors that did not yield activity may have been tested and are not included*.

At least two other CWGTs have been identified based on phenotypes resulting from mutational genetics. XAX1, a XylT that adds xylose side chains to grass arabinoxylans, was identified in a reverse genetics screen focused on GT61 enzymes due to a cell wall xylose deficiency (Chiniquy et al., 2012). XGD1, which adds xylose to the HG backbone, was identified from mutants that were 25% deficient in xylose composition (Jensen et al., 2008; Table 2).

### Omics-Directed GT Selection

The omics-directed GT selection approach bypasses the step of first measuring activities within native plant microsomal enzyme sources or identifying a putative GT from mutant phenotypes. Rather, omics data are analyzed using bioinformatics to predict GTs from genomic sequences prior to recombinant expression and assaying of an enzyme's GT activity. GTs have common features that are conserved across species which may include the presence of transmembrane (TM) domains, conserved motifs, and three-dimensional structural folds (Lairson et al., 2008). Structural features most commonly exploited in bioinformatics approaches have been the type II TM structure and the presence of Asp-X-Asp (DXD) motifs that participate in nucleotide sugar substrate binding in GT-A fold type glycosyltransferases (Sarria et al., 2001; Egelund et al., 2004; Lairson et al., 2008; Qu et al., 2008). Despite being powerful predictive tools, it is important to caution that gene predictions resulting from the use of bioinformatic methods and genomic databases require verification with standard biochemical methods to prove the ascribed enzyme function.

The CAZy database groups putative GTs annotated in genomic databases into families that are predicted to share structural folds and sequence similarities based on their primary amino acid sequences (Lombard et al., 2014). A GT clone collection consisting of over 400 Arabidopsis GT sequences, representing 88% of the putative CWGTs listed in CAZy, has been created to simplify the process of heterologous expression (Lao et al., 2014). Given the limited number of nucleotide sugar donors needed to synthesize all PCW glycosyl linkages, GTs can be screened for activity even without a predicted donor substrate. For example, XGD1 expressed in *N. benthamiana* was incubated with five different radiolabeled UDP-sugars, but only UDP-xylose led to significant levels of radiolabel detected in polymeric products (Jensen et al., 2008; Table 5). The technique of nucleotide sugar suite screening is effective due to the tendency of GTs to hydrolyze specific nucleotide sugar substrates *in vitro*, transferring the glycosyl residue to water in the absence of an exogenously-added acceptor. Therefore, many GT activities can be detected without knowledge of the proper acceptor substrate (Sheikh et al., 2017).

**Table 4:** Mixed-linkage glucan biosynthetic GTs of known function determined by heterologous expression.

**Mixed-linkage glucan (MLG)**							
**Enzyme or family**	**Sugar trans.^a^**	**Activity^b^, ***Product synthesized***^c^**	**GT^d^**	**Acceptor^e^**	**Activity notes**	**Homology/redundancy (Arabidopsis)**	**References**
CSLF	D-Glc	(1,3;1,4)-β-Glc *MLG backbone*	2	Unknown: endogenous acceptors or *de novo* synthesis in Arabidopsis, *N. benthamiana*, and *P. Pastoris*.	Unknown length of MLG backbone products. Varying DP3:DP4 ratios from barley (~1.6:1) and sorghum (1.0:1) CSLF6 orthologs.	MLG not detected in Arabidopsis. 10-member family in *Poaceae*. Activity for CSLF6, Gain-of-function evidence for CSLF2 and 4.	Burton et al., 2006; Vega-Sanchez et al., 2012; Jobling, 2015; Kim et al., 2015; Dimitroff et al., 2016; Little et al., 2018
CSLH	D-Glc	(1,3;1,4)-β-Glc *MLG backbone*	2	Unknown: endogenous acceptors or *de novo* synthesis in Arabidopsis. Gain-of-function evidence only.	Unknown length of MLG backbone products. DP3:DP4 ratio ~3.6 from barley enzyme.	MLG not detected in Arabidopsis. Barley enzyme.	Doblin et al., 2009; Little et al., 2018
CSLJ	D-Glc	(1,3;1,4)-β-Glc *MLG backbone*	2	Unknown: endogenous acceptors or *de novo* synthesis in *N. benthamiana*. Gain-of-function evidence only.	Unknown length of MLG backbone products. DP3:DP4 ratio ~1.3 from barley enzyme.	MLG not detected in Arabidopsis. Barley enzyme.	Little et al., 2018

*Gene annotations, abbreviations, and references are formatted as in Table 1*.

a *Trans. is an abbreviation for transferred*.

b *Activities are listed as simplified descriptions of the monosaccharide transferred. Side chain activities are listed as “initiation” for the first residue attached to the polysaccharide backbone or “addition” for subsequent residues added to an extended side chain. Full activity names include the designation “transferase” and the polysaccharide acceptor, where applicable. As an example, the full activity name of MLG backbone synthesis is β-(1,3;1,4)-glucosyltransferase [β-(1,3;1,4)-GlcT] and the full linkage synthesized is Glcβ-(1,3;1,4)-Glc*.

c *Products synthesized are listed in italics by common names used in the literature, where applicable*.

d *CAZy GT family*.

e *Only acceptors with confirmed activity in the references cited are listed. Other acceptors that did not yield activity may have been tested and are not included*.

**Table 5:** Pectin biosynthetic GTs of known function determined by heterologous expression.

**Pectin**							
**Enzyme or family**	**Sugar trans.^a^**	**Activity^b^, ***Product synthesized***^c^**	**GT^d^**	**Acceptor^e^**	**Activity notes**	**Homology/redundancy (Arabidopsis)**	**References**
GAUT	D-GalA	α-1,4-GalA *Homogalacturonan (HG) backbone*	8	HG backbone oligosaccharides, DP 3–15. *De novo* synthesis from UDP-GalA.	Elongation to HMW polymers >100 kDa. Large rate increase with acceptors DP ≥ 11.	15-member family. Activity for GAUT1:GAUT7 complex, GAUT1, 4, and 11. High MW polymerization detected with GAUT1:GAUT7 complex only.	Sterling et al., 2006; Atmodjo et al., 2011; Amos et al., 2018; Biswal et al., 2018; Voiniciuc et al., 2018a
XGD1	D-Xyl	β-1,3-Xyl side chain initiation on HG backbone *Xylogalacturonan (XGA)*	47	HG backbone oligosaccharide acceptor mix, DP 12–14.	Single addition to HG backbone. Unknown pattern of transfer or regiospecificity.	Activity for XGD1 only. Six additional homologs in GT47 subgroup C, no functions assigned.	Jensen et al., 2008
RRT1	L-Rha	α-1,4-Rha linkage on the non-reducing GalA of RG-I backbone acceptor: [-2)-α-Rha-(1,4)-α-GalA-(1-] *RG-I backbone*	106	RG-1 repeating disaccharide acceptors of DP 5–14 containing GalA on the non-reducing end.	Single addition. GalAT activity needed for disaccharide elongation. Highest relative activity with DP 10 acceptor. DP 3–4 acceptors tested, no activity detected.	Four-member family. Activity for RRT1, 2, 3, and 4 detected.	Uehara et al., 2017; Takenaka et al., 2018
GALS	D-Gal and L-Ara*_*p*_*	β-1,4-Gal side branch elongation of galactans linked to RG-I backbone *RG-I/AG side branch* Secondary activity: Ara*p* termination of β-1,4-Gal side branch, unknown linkage.	92	AG oligosaccharides (β-1,4-Gal), DP 4–7. DP 1–3 acceptors tested, minimal, or no activity detected.	Elongation of DP 5 acceptor to DP ~ 11 detected by carbohydrate gel electrophoresis. High MW polymerization unknown. Acceptors of DP ≥ 5 preferred. Single addition of Ara_p_ prevents further elongation of chain. ~10-fold higher affinity for UDP-Gal over UDP-Ara*p*.	Three-member family. GalT activity for GALS1, 2, and 3. Lower activity/less transfers for GALS2 and 3. Ara*p*T activity for GALS1 only.	Liwanag et al., 2012; Ebert et al., 2018; Laursen et al., 2018
RGXT	D-Xyl	α-1,3-Xyl addition to Fuc in RG-II side chain A	77	L-Fuc monosaccharide.	Single addition to synthesize Xyl-Fuc disaccharide. Full oligosaccharide acceptor containing RG-II backbone and side chain A residues not tested.	Four-member family. Activity for all RGXT1, 2, 3, and 4.	Egelund et al., 2006, 2008; Petersen et al., 2009; Liu et al., 2011

*Gene annotations, abbreviations, and references are formatted as in Table 1*.

a *Trans. is an abbreviation for transferred*.

b *Activities are listed as simplified descriptions of the monosaccharide transferred. Side chain activities are listed as “initiation” for the first residue attached to the polysaccharide backbone or “addition” for subsequent residues added to an extended side chain. Full activity names include the designation “transferase” and the polysaccharide acceptor, where applicable. As an example, the full activity name of HG backbone synthesis is homogalacturonan:α-1,4-galacturonosyltransferase (HG:α-1,4-GalAT) and the full linkage synthesized is GalAα-1,4-GalA*.

c *Products synthesized are listed in italics by common names used in the literature, where applicable*.

d *CAZy GT family*.

e *Only acceptors with confirmed activity in the references cited are listed. Other acceptors that did not yield activity may have been tested and are not included*.

A prominent example of a CWGT discovered through prediction from sequence homology is the β-1,4-GalT activity of GALS1. The GT92 family was proposed following the phylogenetic analysis of a β-1,4-galactosyltransferase from *C. elegans* (Titz et al., 2009). In studying the Arabidopsis GT92 proteins of unknown function, a combination of mutational genetics in which plants had a deficiency in β-1,4-galactan in the cell wall and omics-directed GT selection revealed that the protein later annotated as GALS1 incorporated galactose into β-1,4-galactan acceptors using UDP-Gal as a sugar donor (Liwanag et al., 2012; Table 5). This enzyme was later found to have a secondary activity, in which GALS1 can terminate chain elongation by transfer of an Ara_*p*_ residue onto the same acceptors (Laursen et al., 2018).

The reasoning leading to the discovery of the GalT activity of GALS1 is unlikely to apply to all GT families. The GalT activity of GALS1 was predicted because GT92-family proteins contain β-1,4-galactosyltransferases in other organisms (Liwanag et al., 2012). In contrast, each CAZy GT family is not necessarily limited to a single nucleotide sugar donor. For example, the GT8 family, which contains GAUT family enzymes that transfer GalA from UDP-GalA (Sterling et al., 2006), also contains enzymes that use UDP-Glc (Gibbons et al., 2002), UDP-Gal (Persson et al., 2001), UDP-Xyl (Inamori et al., 2012; Yu et al., 2015), and UDP-GlcNAc sugar donors (Yoko et al., 2003; Chen et al., 2016). Within this single GT family, activities also vary between single-addition linkages, polysaccharide chain polymerization activities, and protein O-linked glycan synthesis. Classification within a CAZy family may only be expected to predict the three-dimensional structural fold and the stereospecificity of the linkage (retention vs. inversion of the anomeric configuration relative to the nucleotide sugar donor) (Lombard et al., 2014).

Taking advantage of the highly upregulated rate of cellulose synthesis in developing cotton fibers, an early use of genomic techniques led to the identification of the cellulose synthase genes based on homology of cDNA clones from cotton (*Gossypium hirsutum*) to the previously-discovered bacterial cellulose synthase from *Acetobacter xylinum* (Pear et al., 1996; Richmond and Somerville, 2000). The cellulose synthase superfamily contains several sub-clades with predicted β-linked polysaccharide synthesis activities, known as the cellulose synthase-like (CSL) gene families (Richmond and Somerville, 2000; Little et al., 2018). Four monosaccharides (d-Glc, d-Gal, d-Man, and d-Xyl) can form β-linked polysaccharide backbones in PCWs. Using a combination of all of the approaches described above, each β-linked hemicellulose (xyloglucan, mixed-linkage glucan, xylan, mannan, and galactomannan) has been assigned to one or more of the CSL families. Monosaccharide composition was not able to directly implicate *csld* mutants in the biosynthesis of a particular hemicellulosic polysaccharide, but incubation of microsomal membranes prepared from *N. benthamiana* leaves transfected with CSLD genes with the four possible UDP-sugars was instrumental to identifying the CSLD family as mannan synthases (Table 3). Enhanced activity from microsomes expressing CSLD5 or co-expressed CSLD2:CSLD3 was only detected with GDP-Man (Verhertbruggen et al., 2011; Yin et al., 2011). Uncertainties remain regarding gene redundancy within the CSLD family, the size of mannan polysaccharides synthesized, and mechanistic details of mannan synthesis. The activity of CSLD is considered not to have been definitively identified (Little et al., 2018). However, current activity assays suggest that the CSLA and CSLD families both encode mannan synthases.

With the mapping of most or all PCW backbone synthesizing enzymes to one or more GT families (conclusive identification of the RG-I backbone polymerizing activity remains to be confirmed), the identity of the GTs that catalyze the synthesis of the more challenging and numerous side-chain linkages remains to be completed. Omics-directed GT selection has provided powerful approaches to identify GTs that synthesize side-chain linkages among the hundreds of putative GTs in plant genomes. XXT1, one of the XylT enzymes that synthesize the X side chain of XG, was identified by prediction due to its grouping in GT34, the same family as the previously-identified fenugreek mannan GalT. The XylT activity was first detected in pea microsomes, and then the XXT enzyme was identified as the only one of 7 expressed GT34 enzymes with XylT activity (Faik et al., 2002; Table 1). The GT77 family containing the RGXT family of four enzymes with XylT activity involved in RG-II side chain A synthesis was identified using a bioinformatics approach aimed at searching for cell wall GTs of previously-unidentified function (Egelund et al., 2004, 2006; Table 5). Coexpression servers were used to predict the activity of the xylan-active GUX family which adds GlcA onto the xylan backbone (Table 2). This GT co-expresses with other xylan biosynthetic enzymes (Mortimer et al., 2010; Rennie et al., 2012).

Omics-based bioinformatic approaches have been particularly fruitful for identifying enzymes related to arabinogalactan protein (AGP) glycosylation. The GT31 family in Arabidopsis contains the GALT and HPGT families that initiate Gal synthesis on hydroxyproline residues, At1g77810 which elongates β-1,3-Gal backbones, and the GalT31 family that elongates β-1,6-Gal side branches (Qu et al., 2008; Basu et al., 2013; Geshi et al., 2013; Ogawa-Ohnishi and Matsubayashi, 2015; Showalter and Basu, 2016; Table 6). The Arabidopsis FUT family, GT37, is unique in that this family contains enzymes that function in the synthesis of different types of PCW polysaccharides and glycoconjugates including xyloglucan and AGPs. Mutational genetics revealed that fucosylation is important for root expansion, and the high expression of FUT4 and FUT6 in roots led to their identification as having AGP-specific FucT activities (Sarria et al., 2001; Tryfona et al., 2014; Table 6).

**Table 6:** Arabinogalactan protein biosynthetic GTs of known function determined by heterologous expression.

**Arabinogalactan protein (AGP)**							
**Enzyme or family**	**Sugar trans.^a^**	**Activity^b^, ***Product synthesized***^c^**	**GT^d^**	**Acceptor^e^**	**Activity notes**	**Homology/redundancy (Arabidopsis)**	**References**
GALT	D-Gal	β-1,4-Gal backbone initiation on AGP hydroxyproline residue *AGP backbone* (initiation only)	31	Synthetic peptide acceptor consisting of 7 or 14 Ala-Hyp [AO] dipeptide repeats. Deglycosylated protein acceptor containing 51 [AO] repeats.	Single addition to initiate AG chain on AGP. Relatively higher activity with synthetic peptides than with deglycosylated protein acceptor. Unknown pattern of transfer or regiospecificity.	Five-member family. Activity for GALT2-6. GALT1 is non-cell wall related.	Basu et al., 2013, 2015a,b; Showalter and Basu, 2016
HPGT	D-Gal	β-1,4-Gal backbone initiation on AGP hydroxyproline residue *AGP backbone* (initiation only)	31	Synthetic peptide acceptor consisting of repeats containing non-contiguous Hyp residues.	Single addition to initiate AG chain on AGP. Relatively higher activity with peptides containing more repeats.	Three-member family. Activity for HPGT1, 2, and 3.	Ogawa-Ohnishi and Matsubayashi, 2015
At1g77810	D-Gal	β-1,3-Gal *AG backbone*	31	β-1,3-Gal disaccharide acceptor.	Single addition to disaccharide synthesizes DP3 products. Continued elongation of AG backbone unknown.	No identified homologs.	Qu et al., 2008
GALT29A	D-Gal	β-1,6-Gal side branch elongation and initiation on β-1,3-Gal backbone *AG side branch*	29	Heterogeneous AGP acceptor mix on a synthetic peptide expressed and glycosylated in *N. benthamiana*, providing acceptor sites for β-1,6-Gal and β-1,3-Gal addition. β1-3-Gal acceptor of MW 25 kDa (~DP150).	Elongation of β-1,3 (backbone) and β-1,6- (side branch) acceptors. Unknown length of elongation or specific acceptor preferences.	No identified homologs.	Dilokpimol et al., 2014
GALT31A	D-Gal	β-1,6-Gal side branch elongation *AG side branch*	31	Heterogeneous AGP acceptor mix on a synthetic peptide expressed and glycosylated in *N. benthamiana*, providing acceptor sites for β-1,6-Gal and β-1,3-Gal addition. β-1,6-Gal DP3 acceptor.	Elongation of β-1,6- (side branch) acceptors. Elongation of DP 3, but not DP 2, acceptors. Unknown length of elongation.	No identified homologs.	Geshi et al., 2013
GlcAT14	D-GlcA	β-1,6-GlcA side chain addition to β-1,3-Gal AG backbone and β-1,6-Gal AG side branches	14	Heterogeneous AGP acceptor mix on a synthetic peptide expressed and glycosylated in *N. benthamiana*. AGP side chain oligosaccharides (β-1,6-Gal) DP 3–11 and backbone oligosaccharides (β-1,3-Gal) DP 5 and 7.	Single addition to both β-1,6 (side branch) and β-1,3 (backbone) linkages within AGP polysaccharides. Unknown pattern of elongation. No activity detected with β-1,3-Gal oligosaccharides DP <5.	Three-member family. Activity for GlcAT14A, B, and C.	Knoch et al., 2013; Dilokpimol and Geshi, 2014
FUT4/FUT6	L-Fuc	α-1,2-Fuc addition to various α-1,3-Ara linked to the β-1,6-Gal AG side branches	37	Non-fucosylated AGP polysaccharide extracted by Yariv reagent from tobacco BY2 cells.	Single addition to L-Ara side chain residues in both β-1,6 (side chain) linkages within AGP polysaccharides. Unknown pattern of elongation.	10-member FUT family. FUT4 and FUT6 are AGP-specific, redundancy unknown. Two known Fuc acceptor sites in AGP.	Wu et al., 2010

*Gene annotations, abbreviations, and references are formatted as in Table 1*.

a *Trans. is an abbreviation for transferred*.

b *Activities are listed as simplified descriptions of the monosaccharide transferred. Side chain activities are listed as “initiation” for the first residue attached to the polysaccharide backbone or “addition” for subsequent residues added to an extended side chain. Full activity names include the designation “transferase” and the polysaccharide acceptor, where applicable. As an example, the full activity name of AGP backbone synthesis is β-1,3-galactosyltransferase (β-1,3-GalT) and the full linkage synthesized is Galβ-1,3-Gal*.

c *Products synthesized are listed in italics by common names used in the literature, where applicable*.

d *CAZy GT family*.

e *Only acceptors with confirmed activity in the references cited are listed. Other acceptors that did not yield activity may have been tested and are not included*.

Recent advances in the production of suitable acceptor substrates with PCW-relevant glycan linkages hold promise for increasing the rate of identifying CWGTs. The purification of heterogeneous acceptor mixes of complex glycans such as AGPs has enhanced detection of GT activity from putative genes identified by omics-directed selection (Xu et al., 2005; Geshi, 2014). The availability of a heterogeneous mixture of AGP oligosaccharide acceptors makes it possible to identify enzyme activity by incubating the expressed enzyme with an acceptor mixture and a wide range of radiolabeled nucleotide sugar donors, followed by the use of structure-specific hydrolases to map the location of radiolabel incorporation. AGP-specific gene families GALT31A, GALT29A, and GlcAT14 were identified using this approach (Geshi et al., 2013; Dilokpimol and Geshi, 2014; Dilokpimol et al., 2014; Table 6). Some uncertainties remain over the functional redundancy of the large number of β-Gal transferase activities that synthesize 1,4; 1,3; and 1,6 AGP linkages (Showalter and Basu, 2016). More precise acceptors will be necessary to understand the length control or regiospecificity of enzymes within these families.

Some acceptors, especially complex polysaccharides such as RG-II with many highly specific side-chain linkages, cannot easily be purified. New bacterial hydrolases capable of catalyzing the cleavage of almost all of the specific linkages within the RG-II side chains have been identified and biochemically characterized (Ndeh et al., 2017). The availability of hydrolytic enzymes allows for the production of acceptor substrates for activity assays, and the specific hydrolysis of enzymatically-formed glycan linkages is a common method to verify the activity of a recombinant enzyme. Additionally, large libraries of glycan epitopes have been synthetically produced that will be useful to define the acceptor substrate specificity of individual GTs (Bartetzko and Pfrengle, 2018). The structural complexity of PCW glycans will require a combination of the above techniques to resolve outstanding questions in cell wall biosynthesis.

### Gain-of-Function Assays

Gain-of-function assays involve the expression of a putative cell wall biosynthetic GT in a plant species that does not normally express the proposed polysaccharide or side-chain linkage. These assays take advantage of differences in cell wall polysaccharide compositions that exist in different plant species or different tissues within a single species. Although gain-of-function assays are a type of heterologous expression method, they result in the *in vivo* synthesis of non-native polysaccharides or side-chain decorations that may be detected by analytical methods. *In vivo* synthesis always occurs within an environment of known and unknown biosynthetic enzymes and machinery. Thus, although gain-of-function assays may provide strong evidence for the function of a putative GT, standard *in vitro* biochemical assays are required to confirm the enzymatic activity.

Gain-of-function methods have led to the detection of several non-native polysaccharides and side-chain decorations in Arabidopsis and tobacco, notably mixed-linkage glucans (MLGs), arabinosylation of xylan, and additional XG side-chains. Mixed-linkage glucans (MLGs) are unbranched, unsubstituted β-glucan chains that contain both 1,3 and 1,4 linkages arranged in a non-repeating but non-random fashion. MLGs are a class of cell wall polysaccharide upregulated in grasses, but are not synthesized by Arabidopsis or tobacco (Doblin et al., 2009). The synthesis of MLG has now been mapped to three different families, CSLF, CSLH, and CSLJ, by overexpression of representative enzymes in either Arabidopsis or tobacco cells, leading to the accumulation of MLG polysaccharides in cell walls (Burton et al., 2006; Doblin et al., 2009; Dimitroff et al., 2016; Little et al., 2018; Table 4). The activity of CSLF6 has separately been confirmed *in vitro* using enzyme expressed in *Pichia* microsomes, further corroborating the usefulness of the now well-established gain-of-function methods typically used to demonstrate MLG synthesis activity (Kim et al., 2015). Similar techniques have recently been developed to study mannan synthesis by accumulation of heteromannans in the cell walls of the non-native host *Pichia pastoris* (Voiniciuc et al., 2019). Xylan structures are different across species, and Arabidopsis has a limited ability to synthesize arabinose-containing side chains (arabinoxylan). A GT61 enzyme that synthesizes arabinoxylan side-chains, XAT1, was discovered by a gain-of-function assay that led to the detection of arabinoxylan in Arabidopsis cell walls (Anders et al., 2012). In addition to the four XG biosynthetic activities verified by *in vitro* assays (Table 1), at least 24 different XG structures have been identified (Pauly and Keegstra, 2016). Gain-of-function and overexpression experiments have contributed to evidence for at least three additional biosynthetic GTs (XUT1, XST, and XDT) resulting in the synthesis of additional side-chain linkages not normally found in Arabidopsis leaf tissues (Pena et al., 2012; Schultink et al., 2013; Zhu et al., 2018).

## Heterologous Expression Systems for Plant Cell Wall GTs

After identifying a putative GT by one, or a combination, of the methods described above, heterologous expression allows for purification and biochemical verification of the proposed activity (Figure 1). Most Golgi-localized GTs are predicted to have either a type II membrane protein structure, with a single TM domain and a catalytic domain that usually faces the Golgi lumen, or a multi-TM domain structure (Oikawa et al., 2013; Kellokumpu et al., 2016). Heterologous expression strategies usually involve either expression of a construct that contains a truncated N-terminus to remove the TM domain or expression of the full-length construct as a membrane-bound protein.

Secretory pathway proteins undergo folding and post-translational modification within the endoplasmic reticulum. Two post-translational modifications important for protein folding are *N*-glycosylation and disulfide bond formation. Protein glycosylation is a major part of the quality control system by which eukaryotic cells prevent misfolded proteins from exiting the ER and entering the later compartments of the secretory system (Xu and Ng, 2015). The ability of eukaryotic expression systems to carry out these post-translational modifications may be necessary for successful protein expression and GT recovery.

### Expression of CWGTs as Soluble Proteins in Bacteria: *Escherichia coli*

*Escherichia coli* is frequently the preferred system for heterologous expression of enzymes due to the relatively inexpensive growth media and minimal training required to express proteins (Kaur et al., 2018). However, *E. coli* has been largely ineffective for the expression of CWGTs, likely due to insufficient protein translational folding and *N*-glycosylation (Welner et al., 2017; Kaur et al., 2018). Some bacteria, notably *C. jejuni*, do have *N*-glycosylation pathways. Efforts have been made to engineer *E. coli* cells with the *N*-glycosylation locus of *C. jejuni* and to use periplasmic-directed expression vectors to assist in the folding of heterologously-expressed eukaryotic proteins (Fisher et al., 2011; Valderrama-Rincon et al., 2012). In general, however, the protein glycosylation pathways in bacteria are not comparable to those of eukaryotic systems and the resulting *N*-glycans do not have similar glycosyl compositions to *N*-glycans typically observed on eukaryotic proteins (Nothaft and Szymanski, 2013). The oxidative environment of the periplasm performs protein folding and disulfide bond formation in *E. coli*, but the system of chaperones and oxidizing enzymes are not capable of properly folding many complex eukaryotic enzymes (Groff et al., 2014; Hatahet et al., 2014).

Few examples of CWGTs have been successfully expressed using *E. coli* and it appears unlikely that optimized expression in *E. coli* will yield the milligram quantities of enzyme needed for structural studies that have been obtained using eukaryotic systems such as HEK293 cells. The xyloglucan xylosyltransferase (XXT)-family enzymes and the AGP enzyme GalT31A are the only examples of CWGTs from which *E. coli* expressed proteins have yielded activity (Chou et al., 2012; Vuttipongchaikij et al., 2012). In one study, 3–7 μg of XXT enzyme was recovered from a 75 mL culture, by purifying enzymes directly from supernatants using glutathione beads (Vuttipongchaikij et al., 2012; Table 1). GalT31A was purified by a similar method, also yielding low microgram amounts (Geshi et al., 2013; Table 6). Another study purified sufficient amounts of protein to compare the kinetic rates of three family members, XXT1, XXT2, and XXT5 (Culbertson et al., 2016; Table 1). A high-throughput study has recently been published in which expression in *E. coli* was attempted for 46 CWGTs. Soluble versions of CWGTs that have been successfully expressed in eukaryotic sources, including IRX10L (Xys1), MUR3, GALS1, and GAUT1, were either not expressed or expressed at very low yields, and none them were purified or assayed for activity (Welner et al., 2017). Large-scale cultures of *E. coli* may only expect to yield, at most, low microgram quantities of soluble protein. One exception is reversibly glycosylated peptide-1 (RGP1) which was able to be expressed with a yield of ~1 mg from a 2 L culture (Welner et al., 2017). However, while RGP1 is classified as a GT75-family enzyme, it does not have a signal peptide or transmembrane sequence (Dhugga et al., 1997), unlike the majority of other Golgi-localized GTs. This difference in topological structure may account for its increased success in *E. coli* expression relative to other GTs tested. RGP1 has also been shown to have a mutase activity that converts UDP-l-arabinofuranose to UDP-l-arabinopyranose (Rautengarten et al., 2011; Welner et al., 2017) and has been suggested to function as part of a xylan synthase complex in species, such as wheat, that synthesize arabinosylated xylan (Jiang et al., 2016). The possibility remains that RGP1 is a multifunctional enzyme with separate glycosyltransferase and mutase functions in cell wall synthesis (Dhugga et al., 1991, 1997). In addition, RGP1 has been proposed to have a potential role in plasmodesmatal transport (Sagi et al., 2005). The lack of success of purifying most CWGTs as soluble enzymes in *E. coli* (Culbertson et al., 2016) is consistent with the known theoretical limitations associated with the recombinant expression of complex eukaryotic proteins in bacterial systems.

### Expression of CWGTs as Soluble Proteins in Eukaryotes: HEK293 Cells

For GTs with a type II membrane structure, eukaryotic systems have been established allowing soluble protein expression via truncated protein constructs. Truncation of the TM domain and coupling with a recombinant N-terminal secretion signal allows eukaryotic hosts to process soluble GTs using the secretory pathway. The human embryonic kidney cell system (HEK293 cells) and a vector library of all known human GTs has been established, resulting in high-yield soluble protein expression (Moremen et al., 2018). Recent efforts have begun to adapt this system for the expression of PCW biosynthetic GTs.

The highest yield of a heterologously expressed CWGT that has been reported was for FUT1 when expressed in two HEK293 cell lines, HEK293F and HEK293S, with protein yields ranging from ~100–120 mg/L cell culture volume (Urbanowicz et al., 2017; Table 1). The enhanced yields from HEK293 cells have made possible the solutions of the first two crystal structures of CWGTs, FUT1, and XXT1 (Urbanowicz et al., 2017; Culbertson et al., 2018; Table 1). HEK293S cells are deficient in the *N*-glycosylation pathway enzyme *N*-acetylglucosaminyltransferase I (GnTI-), resulting in only high mannose-type *N*-glycosylation. Use of this cell type is desirable for X-ray crystallography screening because digestion with Endoglycosidase F1 results in a polypeptide with single GlcNAc residue at each *N*-glycosylation site (Urbanowicz et al., 2017). Xys1 from Arabidopsis and a green algal species (*Klebsormidium flaccidum*), XXT2, and the GAUT1:GAUT7 complex have also been expressed in HEK293F cells in sufficient quantities for detailed enzymatic characterization and kinetics studies (Urbanowicz et al., 2014; Amos et al., 2018; Jensen et al., 2018; Ruprecht et al., 2018; Tables 1, 2, 5).

### Membrane-Based Eukaryotic Protein Expression: *Nicotiana benthamiana* and *Pichia pastoris*

*N. benthamiana* and *P. pastoris* are the two eukaryotic systems most commonly used to express CWGTs. Microsomal membranes enriched for the protein of interest are prepared following transient expression of gene constructs in *N. benthamiana* leaves or in *P. pastoris* cells. Activity can be measured directly in the membrane fraction or the protein of interest can be purified by affinity chromatography following solubilization of membranes. Radiolabeled nucleotide sugar donors are frequently used to distinguish polysaccharide products synthesized *in vitro* from previously-synthesized polysaccharides within the membrane preparation. The main advantage of these systems is the expression of full-length, membrane-bound proteins, which is especially beneficial for the multi-TM-domain containing GT2-family enzymes of the CSL superfamily (Liepman et al., 2005; Cocuron et al., 2007; Yin et al., 2011)

When microsomal membrane preparations are used, the expressed proteins exist in unknown concentrations relative to the entire membrane protein population, which is a heterogeneous mixture of proteins derived from the ER and Golgi of the host organism. Typically, activity assays are completed using an equivalent amount of microsomal membrane protein compared to background controls to account for native biosynthetic activities (Liwanag et al., 2012; Rennie et al., 2012; Basu et al., 2013). Proteins have been purified from these sources that have been able to yield activity, but protein concentration was generally too small to be measured (Liwanag et al., 2012; Rennie et al., 2012), making these systems unsuitable for precise kinetics or x-ray crystallography studies.

## The Biosynthesis of Extended-Chain Backbone Polymers

PCWs contain polysaccharides that have been measured as long-chain polymers consisting of hundreds to thousands of monosaccharide units. Estimation of polysaccharide size requires careful consideration of the source of the cell wall material, structural information potentially lost during the chemical and enzymatic extraction of polysaccharides from heterogeneous plant tissues, and differences in measurement techniques. Size exclusion chromatography (SEC), multiangle light scattering (MALS), nuclear magnetic resonance (NMR), atomic force microscopy (AFM), and mass spectrometry (MS) have been the primary methods by which chain length has been measured. Table 7 summarizes estimated chain lengths of major matrix polysaccharides made by plants, and compares these sizes to the products synthesized *in vitro* by recombinant enzymes.

**Table 7:** Chain length of plant cell wall polysaccharides synthesized *in vivo* and *in vitro*.

**Polysaccharide**	**Extracted from samples synthesized** ***in vivo***				**Synthesized** ***in vitro***			
	**Chain length (kDa)**	**Chain length (degree of polymerization)**	**Method**	**References**	**Chain length (kDa)**	**Chain length (degree of polymerization)**	**Method**	**References**
Xyloglucan (XG)	9–900 Storage XG: >1000	28–2800	SEC	Park and Cosgrove, 2015	Unknown: assumed to be long due to high ratio of 4-linked glucose to terminal-glucose.		Linkage analysis	Cocuron et al., 2007
	90	280	AFM	Park and Cosgrove, 2015				
Xylan	Glucuronoxylan: ~12	93	NMR	Pena et al., 2007	~3–5	~21–34	MALDI-MS	Urbanowicz et al., 2014; Jensen et al., 2018
	Glucuronoxylan: 5–130 Arabinoxylan and complex heteroxylan: 64–380	N/A	SEC	Ebringerová et al., 2005				
Mannan/Glucomannan (GM)	Woody: 1–64 GalMan: 960–1260	~10–400 ~6000–7800	SEC	Ebringerová et al., 2005	Pure mannan: 175 >2000 GM: 64–560, peak 130	~1000 >6000 ~800	SEC	Dhugga et al., 2004; Liepman et al., 2005
	Konjac GM: 1020 250	~6000 ~1500	SEC-MALS	Makabe et al., 2009; Xu et al., 2013				
	~1–4	11–20	MALDI-MS	Lundqvist et al., 2002				
Mixed-linkage glucan (MLG)	>250	>1500	SEC	Carpita and Mccann, 2010	Unknown: products analyzed after lichenase digestion. Long-chain products not measured.		N/A	Dimitroff et al., 2016
Homogalacturonan (HG)	~14–20	81–117	SEC	Yapo et al., 2007	>100	>500	SEC	Amos et al., 2018
	~60	~320	AFM	Round et al., 2010				
	Embedded within RG-I domains: ~1–2	4–10	MS, NMR	Nakamura et al., 2002; Tan et al., 2013				
Rhamnogalacturonan-I (RG-I)	6 (12% of 50 kDa branched polymer) Full polymer: 23–900	~20 N/A	SEC	Yapo, 2011; Shi et al., 2017	Unknown: due to disaccharide backbone, only single addition to acceptor can be detected.		Anion exchange chromatography and tandem MS	Takenaka et al., 2018
	Debranched: 15 Full polymer: 56	~40 N/A	SEC-MALS	Yapo et al., 2007				
Arabinogalactan from RG-I (AG)	Galactans: ~1–8 Arabinans: ~1–27	2–50 2–200	SEC	Yapo, 2011	Galactans: ~1–2	11	TLC and PACE	Laursen et al., 2018
Arabinogalactan protein (AGP)	5–25	30–120	SEC	Ellis et al., 2010	Unknown: product size not analyzed.		N/A	Geshi et al., 2013; Dilokpimol et al., 2014

*Chain length values (kDa and DP) are either stated directly in references, inferred from reported data, or are a range of figures compiled within reviews. If only the kDa or DP value was reported, the other value was estimated from the molecular weight of component monosaccharides. In some cases, the chain length in DP cannot be estimated due to large amounts of heterogeneity and side-chain branching, indicated by N/A. Use of a tilde (~) indicates estimated values and use of a greater-than sign (>) indicates that high molecular weight polymers that elute near the void volume of a size exclusion column are unable to be precisely measured and the reported size is compared to a dextran standard. SEC, size exclusion chromatography; AFM, atomic force microscopy; MALS, multiangle light scattering; MS, mass spectrometry; MALDI, Matrix-assisted laser desorption/ionization; TLC, thin-layer chromatography; PACE, polysaccharide analysis using carbohydrate gel electrophoresis*.

### Estimates of Cell Wall Polymer Chain Length

Current estimates suggest that the longest PCW polymers are XG, mannan, and MLG. All three of these polysaccharides are synthesized by CSL-family GT2 enzymes (Tables 1, 3, 4), indicating a possible connection between chain length and GT domain structure. Size exclusion chromatography has resulted in estimates of XG chain lengths spanning two orders of magnitude (9–900 kDa). Measurements from AFM (90 kDa) are consistent with this backbone polysaccharide being synthesized to high degrees of polymerization *in vivo* (Park and Cosgrove, 2015). However, no current estimate exists for the size of XG or MLG synthesized *in vitro*. Both short-chain and high molecular weight (MW) mannan structures have been isolated (Ebringerová et al., 2005). Whereas pure mannan synthesized *in vitro* by CSLA9 was confined to very large molecular weights, glucomannan eluted with much broader retention volumes (Liepman et al., 2005; Voiniciuc et al., 2019). Synthesis of XG, mannan, and MLG backbones has been measured using microsomal enzyme sources without the addition of exogenous acceptors (Tables 1, 3, 4). In each case, it is unclear whether the heterologously expressed enzymes elongated endogenous acceptors previously synthesized by the plant cells, or if the CSL-family enzymes responsible for these activities are able to initiate chain synthesis *de novo*.

Measurements of HG extracted from plants have suggested a more constrained polymer size. Size exclusion chromatography has suggested a range of 14–20 kDa (degree of polymerization; DP 81–117) (Yapo et al., 2007) for the HG backbone, and HG was measured by AFM as ~60 kDa (DP 320) (Round et al., 2010). Short HG glycans <10 residues are also embedded within RG-I domains, both as complex heteropectins and as part of larger AGP-linked proteoglycans (Nakamura et al., 2002; Tan et al., 2013). HG polysaccharides have been synthesized *in vitro* with chain lengths longer than these expected sizes. GAUT1:GAUT7 appears to be able to synthesize long-chain HG polymers of an indefinite size (> 100 kDa; DP > 500) *in vitro* (Amos et al., 2018). These results suggest that length control, at least for HG, may not be an intrinsic property of CWGTs, and may require more complex mechanisms *in vivo*.

Estimates from *in vivo* synthesized polysaccharides may be high if the extent of branching and size chains are not considered. The RG-I polysaccharide was originally measured as a long chain of 100–1,000 kDa based on SEC of the full polysaccharide (Mcneil et al., 1980; Albersheim et al., 2010). SEC-MALS estimates have measured the debranched GalA-Rha disaccharide backbone of citrus RG-I as 15 kDa, accounting for only 26% of the mass of a full 56 kDa polymer (Yapo et al., 2007). The differences in size reported for RG-I could be explained by the different source materials (sycamore vs. citrus) or the variation in branching patterns. AG side branches associated with RG-I are themselves branched, and the longer estimates (up to DP 200) likely do not represent a single core polysaccharide (Yapo, 2011). AGs associated with AGP proteins are also highly branched with estimates of 5–25 kDa accounting for the entire polymer (Ellis et al., 2010).

### Mechanisms of High Molecular Weight Polysaccharide Elongation

The elongation of long-chain polysaccharides can occur by an enzymatic mechanism in which a single acceptor molecule binds to the active site and undergoes multiple rounds of elongation before release from the enzyme. This mechanism of synthesis is referred to as enzyme processivity. Because the polysaccharide grows progressively longer during processive synthesis, it must slide along the enzyme without becoming fully detached following each monosaccharide transfer (Breyer and Matthews, 2001). Processive mechanisms can be distinguished from distributive (also called non-processive) mechanisms, in which the enzyme releases the polysaccharide chain following each individual monosaccharide transfer.

Processive enzymes tend to have structural elements that enclose the elongating substrate (Breyer and Matthews, 2001). Several mechanisms exist by which a substrate can be physically constrained within an enzyme active site, preventing diffusion of the product into the surrounding environment. Some glycosyl hydrolases retain their polysaccharide substrates within a tunnel abundant in aromatic amino acyl residues that interact with the sliding carbohydrate chain (Beckham et al., 2014). DNA polymerases form multi-enzyme complexes with sliding clamp enzymes that maintain the DNA-enzyme interaction (Breyer and Matthews, 2001).

The crystal structure of the bacterial cellulose synthase BcsA demonstrates one of the clearest examples of a mechanism for a processive GT. The cellulose synthases, which are CAZy GT2-family enzymes, have multiple TM domains that form a pore through which the growing polysaccharide chain translocates. BscA accommodates a cellulose chain 10 glucose residues in length, spanning from the cytosolic GT domain to the periplasmic pore exit (Morgan et al., 2013). This structural motif, common to GT2-family enzymes, shares homology with other enzymes that synthesize secreted or extracellular matrix polysaccharides, including chitin and hyaluronic acid (Bi et al., 2015).

Notably, all of the CSL-family proteins implicated in the synthesis of PCW β-1,4-glucans (XG, heteromannan, and MLG), are GT2-family enzymes and are predicted to share this multi-TM domain structure (Davis et al., 2010). In contrast, many other GTs have type II membrane domain structures containing only a single TM domain and do not have a similar translocation pore/tunnel. Xys1 is a rare example that has no predicted TM domain (Smith et al., 2017). Xys1 and the type II membrane complex GAUT1:GAUT7 synthesize xylan and HG using distributive (non-processive) mechanisms (Urbanowicz et al., 2014; Amos et al., 2018). Xylan and HG are both long-chain polysaccharides (Table 7), suggesting that processivity is not a requirement for the synthesis of extended polymeric glycans. On the basis of the membrane structure of the biosynthetic CWGTs, RG-I and AGP may also be predicted to be elongated by non-processive mechanisms because the identified GTs are also type II transferases and are not predicted to have multi-TM domains that would serve as translocation pores for processive synthesis.

### Preference for Long-Chain Acceptors—the Two-Phase Mechanism

It has been frequently observed that CWGTs assayed *in vitro* either require acceptors above a certain minimum chain length or show large activity increases toward longer acceptors. These rate increases are often measured with oligosaccharides of a DP 5 to 10 (Tables 1–3, 5). The relevance of increased activity with longer-chain acceptors to enzyme mechanism has been most thoroughly studied in HG elongation by GAUT1:GAUT7 (Table 5). Relative to a DP 7 acceptor, DP 11 acceptors are elongated at a ~6-fold higher rate. DP 11 acceptors also have a ~7-fold higher affinity, as estimated by the K_M_ value measured in Michaelis-Menten kinetics assays. Enzyme kinetics analyses support a comparison of enzymes by combining information on the rate of synthesis (k_*cat*_) with the affinity of the enzyme for a substrate (*K*_M_) to yield a value known as the specificity constant (k_*cat*_/*K*_M_), which is a measure of the catalytic efficiency of a reaction (Copeland, 2000). For GAUT1:GAUT7, the catalytic efficiency of a DP 11 HG acceptor is > 45-fold higher than for that of a DP 7 HG acceptor. The presence of a lag phase during elongation of DP 7 HG acceptors means that the initial-rate preference for DP 11 acceptors is possibly much greater than this value (Amos et al., 2018).

Earlier measures of HG synthesis using solubilized tobacco microsomes were unable to detect elongation of acceptors with a DP <10 (Doong and Mohnen, 1998). Similarly, solubilized *P. axillaris* microsomes detected some elongation of acceptors DP 5–10, but large rate increases were observed with DP ≥ 12 acceptors (Akita et al., 2002). The elongation of short-chain acceptors and the *de novo* initiation of new HG chains were able to be detected *in vitro* using purified GAUT1:GAUT7 enzyme, but with low initial rates of synthesis (Amos et al., 2018). Microsomal sources did not detect these activities, which was likely due to the relatively low enzyme purity and activity measurements achievable using the semi-purified membranes.

There are several other examples in which CWGTs have a minimum acceptor size. XXT1 transfers Xyl to XG backbone acceptors of DP > 4 (Table 1). Activity is increased ~6-fold using DP 6 acceptors relative to DP 5 acceptors (Faik et al., 2002; Cavalier and Keegstra, 2006). The fenugreek GalT that transfers side chain Gal monosaccharides onto mannan backbones has a similar ~5-fold increase in activity with DP 6 acceptors compared to DP 5 acceptors, but no activity for DP 1–4 oligosaccharides (Edwards et al., 1999; Table 3). GUX1 transfers GlcA to xylan backbone acceptors with a preference for DP 6 oligosaccharides, which is a ~10-fold increase over DP 2 acceptors (Rennie et al., 2012; Table 2). GalS1 likewise prefers to elongate galactan acceptors with a DP ≥ 5, and the same galactosyltransferase activity from mung bean microsomes was shown to be ~3-fold greater for DP 7 acceptors than DP 5 acceptors (Ishii et al., 2004; Liwanag et al., 2012; Table 5). Due to limited availability and the insolubility of some classes of oligosaccharide acceptors, assay of CWGTs using acceptors of varying DPs remains to be performed in many cases.

**Figure 2:**
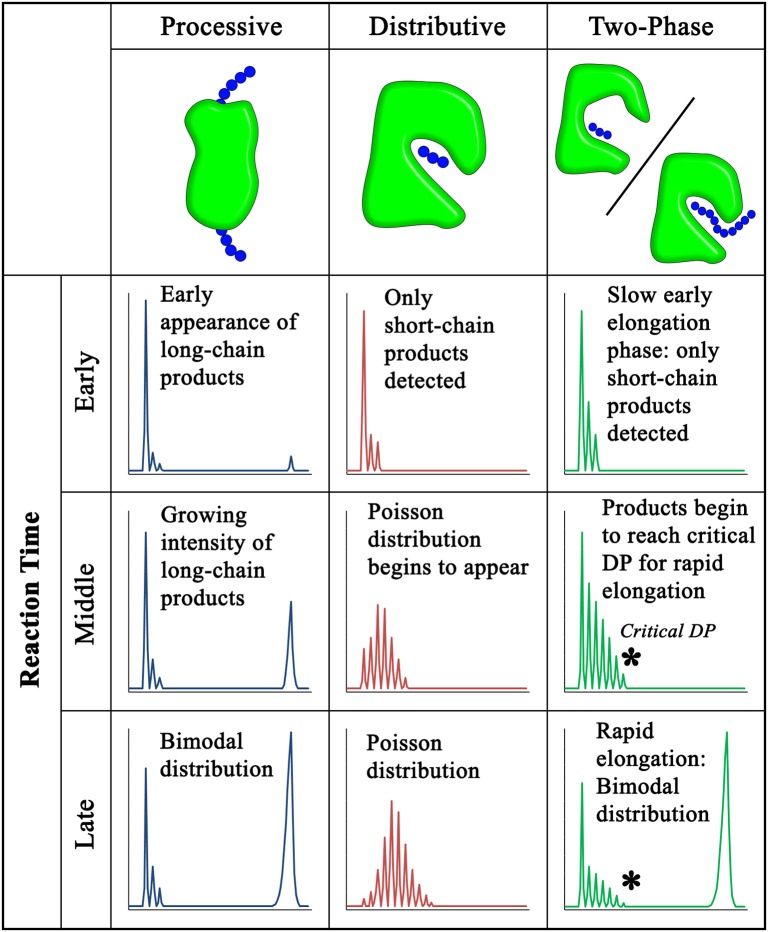
Polysaccharide chain elongation patterns for processive, distributive, and two-phase distributive glycosyltransfer. Glycosyltransferases synthesize polysaccharides by different mechanisms that result in characteristic product profiles over the progress of an elongation reaction. The hypothetical product profiles shown correspond to the elongation of a short-chain acceptor (left) into longer-chain products (right). Three panels are shown for each proposed mechanism corresponding to the early, middle, and late stages of reaction progress. Product profiles are representative of several methods that are used to obtain chain length information, including HPLC, MALDI-MS, and polyacrylamide gel electrophoresis. In processive elongation, formation of an enzyme-substrate catalytic complex that is maintained through many rounds of monosaccharide transfer leads to a bimodal distribution of low and high MW products (Levengood et al., 2011; Raga-Carbajal et al., 2016). In distributive elongation, dissociation of the acceptor substrate following each round of addition and the lack of acceptor length bias leads to a Poisson distribution of products over time (Keys et al., 2014; Urbanowicz et al., 2014). A two-phase distributive mechanism accumulates short-chain products during the early phase of the reaction followed by rapid elongation of high MW polysaccharides resulting from large increases in catalytic efficiency for acceptors with chain lengths longer than a “Critical DP” (Vionnet and Vann, 2007; Amos et al., 2018). The product distribution of a two-phase distributive mechanism can resemble the bimodal distribution observed by processive glycosyltransferases. Chain length analysis may be suggestive of a particular elongation mechanism, but processivity cannot necessarily be inferred without direct evidence, such as the multi-TM translocation pore found in crystal structures of the BcsA:BscB cellulose synthase complex and proposed for other GT2-family structures (Morgan et al., 2013; Bi et al., 2015).

These observations are consistent with a proposed two-phase kinetic mechanism which may be common to CWGTs, in which longer-chain acceptors are elongated at significantly increased rates compared to acceptors below a certain critical DP (Amos et al., 2018). The consequence of two-phase elongation is that elongation of short-chain acceptors may not be detected in some assays because the transfer requires longer incubation times or occurs below the detection limit. Although all of the above-mentioned cell wall-related GTs with preference for longer acceptors appear to be distributive, two-phase elongation is also a feature of bacterial GT2 enzymes, which are known to be processive (Forsee et al., 2006; Levengood et al., 2011). The expected polysaccharide elongation patterns for the three mechanisms of chain elongation: processive, distributive, and two-phase distributive glycosyltransfer are depicted in Figure 2. Chain elongation analysis is integral to determining the mechanisms used by CWGTs to synthesize the full-length polysaccharides deposited into mature cell walls. Without structural data, elongation analysis is the only method to distinguish between these three categories of GT mechanism.

### Limitations of Chain Length Estimation by Size Exclusion Chromatography

Although SEC is a common method used for measuring polysaccharide size, it may result in over-estimation of chain length. Polysaccharides are generally compared to commercially available dextran standards. Differences in polysaccharide stiffness relative to dextrans and the non-globular conformation of polysaccharides may cause these methods to report inaccurate molecular weights (Harding et al., 1991). Size exclusion may also misrepresent polysaccharide length due to the potential for aggregation of polymers or changes to the particle conformations resulting from substitution and decoration (Mort et al., 1991; Park and Cosgrove, 2015).

Glucuronoxylan was estimated as having an average chain length of DP 93 using NMR spectroscopy based on the ratio of reducing end to internal signals, corresponding to a molecular weight of ~12 kDa. The same polysaccharide elutes at a position larger than a 70 kDa based on dextran standards (Pena et al., 2007). This polysaccharide provides an example in which size exclusion may over-estimate the size of a polymer by at least 5-fold. Additional SEC-based molecular weight measurements of glucuronoxylan ranged from 5 to 130 kDa (Ebringerová et al., 2005).

Depending on the standards used in a given experiment, pectic polysaccharides synthesized *in vitro* have been observed to elute at retention volumes larger than 100 kDa and even 500 kDa compared to on dextran standards (Doong et al., 1995; Sterling et al., 2001; Amos et al., 2018). Many commonly-used columns may have limited resolving power at these high MW, resulting in dextran standards >100 kDa tending to elute with overlapping chromatographic patterns and peaks that resolve near the column excluded volume. Under donor substrate-limiting conditions, in which GAUT1:GAUT7 synthesizes HG polymers of ~DP 30–50 (~5–9 kDa) as indicated by individual bands on polyacrylamide gels, the same donor-limited polysaccharides eluted at intermediate sizes between 12 and 50 kDa based on dextran standards (Amos et al., 2018), again indicating that these cell wall polymers appear larger by size exclusion analysis than when measured by other approaches.

### Solubility Limitations of Long-Chain Polysaccharides

The ability to detect high MW products *in vitro* can be hindered by issues with the solubility of longer-chain oligosaccharides. The β-1,4-glucan backbone of XG has minimal solubility above DP 6 and the β-1,4-mannan backbone of mannan is insoluble above DP 8. These insoluble polymers are synthesized by GT2-family CSL enzymes. However, XG extracted from plant cells is soluble due to the presence of side-chain decorations that reduce the amount of intermolecular hydrogen bonding, distinguishing XG from the glucan chains of cellulose microfibrils (Yuguchi et al., 2005). We propose that a likely purpose of the multi-TM domain structure that confers processivity on these transferases is to protect these polysaccharides against aggregation during elongation before other enzymes in the pathway can add side chain decorations (or in the case of cellulose, to enable controlled fiber formation).

The structures of glycosyl hydrolases suggest a connection between processivity and insoluble crystalline polysaccharides, represented by cellulose and chitin (Vaaje-Kolstad et al., 2013). The bacteria S*erratia marcescens* has three GH18 chitinases, ChiA, ChiB, and ChiC. Both ChiA and ChiB have deep clefts for binding to a chitin chain and display higher levels of processivity in activity assays, which is consistent with the hypothesis that processive enzymes enclose their substrates within tunnels. The non-processive chitinase ChiC does not have a similar deep cleft lined with aromatic residues to facilitate processive sliding. Accordingly, ChiC hydrolyzes the soluble form of chitin, chitosan, at higher rates. The structural and activity differences within this family of chitinases suggest that GH enzymes evolved processivity to enhance activity on chains detached from insoluble crystalline polysaccharides, although it comes with the cost of sacrificing enzymatic efficiency (Horn et al., 2006; Vaaje-Kolstad et al., 2013).

## Enzymatic Rates and Kinetic Analyses

Recombinant expression of soluble CWGTs allows for the measurement of reaction rates, which is effectively impossible to accurately measure in microsomal membrane fractions where the enzyme of interest is an unknown percentage of the total protein. Measurement of reaction rates will advance the understanding of how these low-abundance enzymes are able to promote the development of tissues containing large masses of polysaccharides. Additional questions related to the rates of GT activity include how plant cells balance biosynthesis with degradation and remodeling activities, how well-different GTs compete for a limited supply of nucleotide sugar donors within a biosynthetic compartment, and how the rates of backbone synthesis compare to side-chain decoration. The CWGTs with measured reaction rates are summarized in Table 8.

**Table 8:** Enzymatic rates and reaction kinetics of plant cell wall polysaccharides synthesized *in vitro*.

**Enzyme**	**Variable substrate**	**Saturating substrate**	**Donor conc. (μM)**	**Acceptor conc. (μM)**	***K*_M_^a^ variable substrate (μM)**	**Rate^b^ (pmol/min)**	***k*cat^c^: Turnover number (s^**−1**^)**	**Enzyme amount^d^**	**Reaction time (min)**	**References**
**BACKBONE (POLYMERIZING) TRANSFERASES**										
Xys1	Xylohexaose Acceptor	UDP-Xyl Donor	800	0–4000	1.17	0.39	0.0004	15.8 pmol	120	Urbanowicz et al., 2014
GAUT1: GAUT7	UDP-GalA Donor	HG mix DP 7–23 Acceptor	5–2000	100	151 ± 10.6	165.4 ± 3.4	0.92 ± 0.02	3 pmol	5	Amos et al., 2018
	HG DP 7–23 mix Acceptor	UDP-GalA Donor	1000	0.01–50	0.8 ± 0.1	359.0 ± 10.2	1.99 ± 0.06	3 pmol	5	
	HG DP11 Acceptor	UDP-GalA Donor	1000	0.01–100	1.4 ± 0.2	705.0 ± 53.9	3.92 ± 0.3	3 pmol	5	
	HG DP7 Acceptor	UDP-GalA Donor	1000	0.01–100	10.0 ± 1.4	109.8 ± 3.7	0.61 ± 0.02	3 pmol	30	
GalS1	UDP-Xyl Donor	Galactopentaose Acceptor	0–500	50	142 ± 12	656 ± 42	N/A	25 μg microsomal protein	15	Laursen et al., 2018
	UDP-Ara*p* Donor	Galactopentaose Acceptor	0–3000	50	1057 ± 222	454 ± 51	N/A	25 μg microsomal protein	60	
**SIDE-CHAIN TRANSFERASES**										
XXT1	UDP-Xyl Donor	Cellohexaose Acceptor	0–4000	1000	490 ± 40	509.25 ± 14.25	0.11 ± 0.003	75 pmol	30	Culbertson et al., 2016
XXT2	UDP-Xyl Donor	Cellohexaose Acceptor	0–4000	1000	640 ± 90	373.5 ± 17.25	0.083 ± 0.004	75 pmol	30	Culbertson et al., 2016
XXT5	UDP-Xyl Donor	Cellohexaose Acceptor	0–4000	1000	4800 ± 330	166.5 ± 74.75	0.01 ± 0.004	287.5 pmol	60	Culbertson et al., 2016
FUT1	GDP-Fuc Donor	XXLG Oligosaccharide Acceptor	0–400	750	25.32 ± 4.2	14.1 ± 0.2	0.063 ± 0.0008	3.7 pmol	20	Urbanowicz et al., 2017
	XXLG Oligosaccharide Acceptor	GDP-Fuc Donor	200	0–800	201 ± 12.0	N/A	N/A	3.7 pmol	20	
GUX1	UDP-GlcA Donor	Xylohexaose Acceptor	10–3000	400	165 ± 25	N/A (arbitrary units)	N/A	100 μg microsomal protein	60	Rennie et al., 2012

*All units listed in this table are standardized from reported data*.

a *K_M_ values are converted to μM if reported in mM*.

b *Rates are converted to pmol/min per enzyme amount listed in table from reported data*.

c *k_cat_ values are converted to transfers/second per enzyme molecule (s^−1^) from reported data*.

d *Enzyme amounts are converted into pmol if reported in ng using the predicted molecular weights of full-length or truncated proteins. Microsomal protein cannot be used to calculate kcat values due to an unknown concentration of target protein within microsomes. Calculations used to standardize reported values are explained with the original values from each reference in Table S1*.

*Chain length values (kDa and DP) are either stated directly in references, inferred from reported data, or are a range of figures compiled within reviews. If only the kDa or DP value was reported, the other value was estimated from the molecular weight of component monosaccharides. In some cases, the chain length in DP cannot be estimated due to large amounts of heterogeneity and side-chain branching, indicated by N/A. Use of a tilde (~) indicates estimated values and use of a greater-than sign (>) indicates that high molecular weight polymers that elute near the void volume of a size exclusion column are unable to be precisely measured and the reported size is compared to a dextran standard. SEC, size exclusion chromatography; AFM, atomic force microscopy; MALS, multiangle light scattering; MS, mass spectrometry; MALDI, Matrix-assisted laser desorption/ionization; TLC, thin-layer chromatography; PACE, polysaccharide analysis using carbohydrate gel electrophoresis*.

Although published kinetic rates are limited to a small number of CWGTs, a consistent observation is that the turnover rates are slow relative to other classes of enzymes. Popular textbooks depict turnover numbers for enzymes ranging from 0.5 to 600,000 s^−1^, with most enzymes catalyzing 100 or more events per second (Berg et al., 2002). CWGTs, however, typically function at a relatively low rate near or <1 s^−1^ (Table 8). Analysis of published kinetic rates from several thousand enzymes led to the conclusion that most enzymes function at moderate rates, with the median <10 s^−1^ (Bar-Even et al., 2011). Unless the minimalistic conditions used in assays of CWGT activity do not sufficiently replicate proper conditions to measure full enzyme activities, current data suggest that CWGTs are likely to fall into this category of “moderately efficient” enzymes with low enzymatic rates. These kinetic rates, measured using purified enzymes, are strikingly similar to an earlier calculation of microsomal mannan synthase activity. Based on estimation that the GT of interest constituted only 0.02% of total membrane protein, the calculated turnover number was also 1 s^−1^, giving credibility to earlier observations that substantial amounts of cell wall polysaccharides can be deposited over time despite the low polypeptide abundance and low kinetic rates of the biosynthetic machinery (Sandhu et al., 2009).

Many CWGTs exist in multi-gene families. To the limited extent where the enzyme activities of different members of the same gene family have been tested, enzymes catalyze the same apparent activity at different rates. XXT5 was found to have 8 to 10-fold lower catalytic rates than other XXT family members (Culbertson et al., 2016; Table 1), which could be a common trend among multi-gene families (Table 8). The reason for these different activities from members of the same gene family is not presently understood. However, the possibilities include expression in cellular subcompartments that have different substrate concentrations, the production of glycans of different abundance and chain length, or to separately control initiation and elongation activities.

The apparently slow rates of CWGTs may result in false-negative recovery of activity due to expression below the detection limit. In two separate studies of XXT-family enzymes, both using *E. coli* as an expression host, activity for XXT5 was only detected in the study which used a 26-fold higher enzyme concentration (Chou et al., 2012; Culbertson et al., 2016; Table 1). In those cases where heterologous expression is in a plant system, background activity can make it difficult to conclude whether the expressed enzymes are active. Inconsistent results have been reported in GUX family enzymes, where activity for GUX3 was detected after overexpression in tobacco BY2 cells (Table 2) but not in *N. benthamiana* microsomes (Lee et al., 2012a; Rennie et al., 2012). It remains uncertain whether GUX3 is an active xylan glucuronosyltransferase or rather a non-catalytic complex member, as has been proposed (Rennie et al., 2012).

Kinetic analysis was central to modeling the proposed two-phase mechanism by which GAUT1:GAUT7 initiates polysaccharide synthesis at a slower rate than the rate of elongation of longer-chain polymers (Amos et al., 2018; Tables 5, 8). This result explained why in earlier measurements of HG synthesis from tobacco microsomes, only HG acceptors of DP ≥ 10 were found to enhance activity above basal levels in 2-min reactions (Doong and Mohnen, 1998). Elongation of differently-sized acceptors may not necessarily be assumed to be detectable on the same time scales and are likely to require extended kinetic studies accounting for the possibility of reaction phases that have non-linear initial rates. For some polymers, studying the full reaction cycle of polysaccharide synthesis may be limited given the insolubility of longer synthesis products, primarily the unbranched homopolymers with β-1,4 linkages, as discussed above.

Slower rates may also be indicative of the necessity of a GT to function with complex partners for full activity. Because Xys1 is likely to function in a complex, the reported rate is likely to under-estimate xylan backbone synthesis rate given that the full catalytic tertiary structure may not be present when the single enzyme is expressed (Table 2). In asparagus, co-expression of IRX10 (homolog of Arabidopsis Xys1) with homologous proteins IRX9 and IRX14 led to higher activity and longer chain length products, suggesting the potential for enhanced activity as a full xylan synthase complex (Zeng et al., 2016; Table 2). GAUT1 was also found to elongate products to a smaller chain length without GAUT7 (Amos et al., 2018; Table 5). More detailed studies of individually expressed and co-expressed CWGTs may present a relationship between enzymatic rates and the chain length of products synthesized *in vitro*.

The kinetic measurement of GALS1 provides an example of a bifunctional enzyme that may have roles in both chain elongation and termination (Laursen et al., 2018; Tables 5, 8). The potential for a single CWGT to transfer monosaccharides from two different nucleotide sugar substrates underscores the usefulness of screening GT activity with all possible nucleotide sugar substrates because a particular GT may not be limited to a single donor substrate. GALS1 has a 7.4-fold lower *K*_M_ for UDP-Xyl than for UDP-Ara_p_ (Laursen et al., 2018), suggesting a preference for galactan chain elongation over chain termination that may be a mechanism of controlling chain length directly through the differences in enzyme affinity for these two substrates. *K*_M_ is not a direct measure of the equilibrium dissociation constant of an enzyme for its substrate, but *K*_M_ values can be interpreted as a relative measure of substrate affinity (Copeland, 2000).

Preliminary evidence attributes rates to cellulose synthesis that are similar to the synthesis of matrix polysaccharides such as HG (Tables 5, 8). CesA8 from hybrid aspen was expressed in *P. pastoris* cells and synthesized β-1,4-Glc cellulose backbone polymers. However, since kinetic assays were reported in units of relative activity, the results cannot be compared to the CWGTs discussed here (Purushotham et al., 2016). A bacterial cellulose synthase, however, was reported to have a turnover number of 2.9 s^−1^ (Du et al., 2016). Cellulose synthase proteins, like many matrix GTs, function in heteromeric complexes *in vivo*, and the enhancement of activity resulting from complex formation remains to be studied.

## Heterologous Expression of Complexes

Eukaryotic GTs commonly form homo- and heterocomplexes. At least 25 heteromeric GT complexes have been identified from plants, mammals, and yeasts (Kellokumpu et al., 2016). GT complex formation may serve many functions. Several of the most relevant functions are enumerated below (adapted from Kellokumpu et al., 2016):

1. Enhanced activity: Quantitative activity measurements detect a fold-change difference when two or more GTs are co-expressed, or activities cannot be detected without co-expression of an interacting partner.
2. Substrate channeling: Glycosylation is a non-template driven process, and organization of GTs into complexes can protect a glycan from the action of competing enzymes.
3. Golgi localization and protein folding: Some Golgi-localized GTs do not have TM domains and require interaction partners for native protein folding, membrane anchoring, and/or targeting and retention in the proper Golgi sub-compartment.

Complex formation among CWGTs was reviewed previously, and seven proven and putative CWGT hetercomplexes were identified including GAUT1:GAUT7, ARAD1:ARAD2, RGP1:RGP2, and RGP5, the wheat xylan synthase complex, CSLC4:XXT, IRX9:IRX14, and CSLD2:CSLD3 (Oikawa et al., 2013). Several new discoveries have contributed to the effort to define the functional roles of these complexes. Previously, the XG biosynthetic enzyme CSLC4 was discovered to interact with XXT-family enzymes (Chou et al., 2012). Recent studies have demonstrated that all of the enzymes that synthesize the four major monosaccharide linkages in XG (CSLC4, XXT, MUR3, XLT2, and FUT1) interact in close proximity, possibly organizing into a multi-enzyme XG biosynthetic heterocomplex organized around CSLC4 (Chou et al., 2015).

Xylan synthase complexes have now been identified in two species, wheat, and asparagus. The previously-purified wheat xylan synthase complex, with XylT, GlcAT, and AraT activities, was mapped to six individual genes, including enzymes from the GT43 (homolog to IRX14), GT47 (homolog to IRX10), and GT75 (homolog to RGP1, the UDP-arabinopyranose mutase) families (Zeng et al., 2010; Jiang et al., 2016). In contrast, the asparagus xylan synthase complex contains subunits that are homologous to the three families of proposed Arabidopsis xylan synthase enzymes, IRX9, IRX10, and IRX14 (Zeng et al., 2016). Two of the enzymes that elongate galactan backbone and side branches in AGPs (GalT29A and GalT31A) may also interact as a heterocomplex (Dilokpimol et al., 2014).

### Functional Roles of Plant Cell Wall GT Complexes

Following the above numbering scheme for three proposed roles of GT complexes, contributions of CWGT studies to the functions of GT complexes are presented below:

1. Enhanced activity: Co-expression of GAUT1 with GAUT7 leads to the synthesis of high MW HG polysaccharides. GAUT1 expressed alone has a minimal ability to elongate HG acceptors (Amos et al., 2018). Expression of IRX10 leads to minimal elongation of xylan acceptors. Both the highest activity and the longest products were obtained when all three complex members (IRX9, IRX10, and IRX14A) were co-expressed (Zeng et al., 2016). In each case, it is not yet known whether the shorter elongation ability is due to a general decrease in activity or if complex formation creates a domain structure that enhances the ability of GAUT1:GAUT7 or IRX9:IRX10:IRX14 to elongate longer-chain polymers.
2. Substrate channeling: Each enzyme in the XG biosynthesis pathway has a sequential activity (Table 1). The solubility of the glucan backbone is strongly impaired above DP 6, but branching of XG by decoration with Xyl, Gal, and Fuc side-chain residues enhances solubility (Scheller and Ulvskov, 2010). CSLC4 is a multi-TM pore-forming enzyme that extrudes a growing glucan chain into the Golgi lumen (Davis et al., 2010), and complex formation may allow each enzyme to remain in close proximity so that side-chain residues are quickly added before the glucan backbone reaches a chain length at which it becomes insoluble.
3. Golgi localization and protein folding: Co-expression of complex members in both the wheat and asparagus xylan synthase complexes was required for full Golgi localization and export from the ER (Jiang et al., 2016; Zeng et al., 2016). Complex assembly in the ER may be a step in the eukaryotic quality control pathway that prevents misfolded proteins, or in this case, unassembled complexes, from progressing further in the secretory pathway. In the xylan synthase complex, IRX10 lacks a TM domain, and requires interaction with IRX9 and IRX14 for Golgi retention (Zeng et al., 2016). In the HG biosynthetic complex, GAUT1 loses its TM domain *in vivo* after truncation and requires GAUT7 for Golgi retention (Atmodjo et al., 2011). Expression of GAUT1 without GAUT7 results in large amounts of truncation products, high MW aggregates, and GAUT1 homocomplexes, which also supports the notion that co-expression is a necessary part of the folding and quality control process (Amos et al., 2018).

## Conclusions

Molecular descriptions of PCW biosynthesis have advanced over the past 20 years due to the successful development of heterologous expression systems allowing the purification and *in vitro* assay and characterization of biosynthetic genes. At least 26 independent glycosyltransferase activities have been identified corresponding to the synthesis of cell wall polysaccharide backbone and side-chain linkages. Many of these activities are associated with multi-gene families, leading to the potential for understanding the functions of hundreds of biosynthetic genes. Recent successes with expression in HEK293 cell cultures have provided the first examples of large-scale purification of CWGTs for biochemical characterization and crystallographic studies. For the first time, questions related to the mechanisms of biosynthesis and length control of polysaccharides can be investigated, and homogenous cell wall polysaccharides can be synthesized and isolated for the study of their structural properties. Matrix polysaccharides contribute to the complex changes in cellular size, shape, mechanical strength, and adhesion that occur during plant development. The potential for the control of cell wall biosynthesis makes the cell wall biosynthetic GTs promising targets for genetic manipulation to enhance commercially desirable traits of fruits and other plant-derived products.

## Author Contributions

RA conceived and wrote the review and manuscript. DM edited the review and manuscript. RA and DM finalized the manuscript.

### Conflict of Interest Statement

The authors declare that the research was conducted in the absence of any commercial or financial relationships that could be construed as a potential conflict of interest.
