# Supplementary material for: Critical Review of Plant Cell Wall Matrix Polysaccharide Glycosyltransferase Activities Verified by Heterologous Protein Expression
Source: Front Plant Sci. 2019 Jul 16;10:915. doi: 10.3389/fpls.2019.00915 (PMC6646851; doi:10.3389/fpls.2019.00915)
Supplement: Supplementary file 1 [file Table_1.DOCX]

**Table S1. Data from references used to calculate enzymatic rates in Table 3.** Differences noted here include reported data in references using units that are different from those listed in Table 3 and conversion factors used to calculate entries in columns not listed in the original references.

| Enzyme | Notes | Reference |
| --- | --- | --- |
| Xys1 | Activity is reported as *V*_max_ = 0.39 pmol UDP min^-1^. Reactions contain 15.8 pmol enzyme. Calculated turnover number = 0.0004 s^-1^. | (Urbanowicz et al., 2014) |
| GAUT1:  GAUT7 | Reactions contain 100 nM enzyme in 30 µL volume. Calculated enzyme amount = 3 pmol per reaction. | (Amos et al., 2018) |
| GalS1 | Reactions contain 1 µg µL^-1^ total microsomal protein in 25 µL volume. | (Laursen et al., 2018) |
| XXT1 | Reactions contain various UDP-Xyl concentrations. The range of values tested is not reported and are assumed to be 0-4000 µM from Figure 3 in (XXT5 2016). Turnover number (*k*_cat_) is reported as 6.79 ± 0.19 min^-1^. Calculated *k*_cat_ = 0.11 ± 0.003 s^-1^. Reactions contain 3 µM XXT1 in 25 µL volume. Calculated enzyme amount = 75 pmol. Calculated activity in pmol/min = 509.25 ± 14.25. | (Culbertson et al., 2016) |
| XXT2 | Reactions contain various UDP-Xyl concentrations. The range of values tested is not reported and are assumed to be 0-4000 µM from Figure 3 in (XXT5 2016). Turnover number (*k*_cat_) is reported as 4.98 ± 0.23 min^-1^. Calculated *k*_cat_ = 0.083 ± 0.004 s^-1^. Reactions contain 3 µM XXT1 in 25 µL volume. Calculated enzyme amount = 75 pmol. Calculated activity in pmol/min = 373.5 ± 17.25. | (Culbertson et al., 2016) |
| XXT5 | Reactions contain various UDP-Xyl concentrations. The range of values tested is not reported and are assumed to be 0-4000 µM from Figure 3 in (XXT5 2016). Activity (*k*_cat_) is reported as 0.58 ± 0.26 min^-1^. Calculated *k*_cat_ = 0.01 ± 0.004 s^-1^. Reactions contain 11.5 µM XXT1 in 25 µL volume. Calculated enzyme amount = 287.5 pmol. Calculated activity in pmol/min = 166.5 ± 74.75. | (Culbertson et al., 2016) |
| FUT1 | Turnover number (*k*_cat_) is reported as 3.8 ± 0.05 min^-1^. Calculated *k*_cat_ = 0.063 ± 0.0008 s^-1^. Reactions contain 200 ng enzyme in 5-10 µL volume. Calculated enzyme molecular weight = 54.4 kDa based on FUT1 sequence, residues 81-558. Calculated enzyme amount = 3.7 pmol. Calculated activity in pmol/min = 14.1 ± 0.2. | (Urbanowicz et al., 2017) |
| GUX1 | Purified GUX1 protein of an unknown concentration corresponding to 100 µg of microsomal protein was assayed. | (Rennie et al., 2012) |
